# Time-consistent robust investment-reinsurance strategy with common shock dependence under CEV model

**DOI:** 10.1371/journal.pone.0316649

**Published:** 2025-02-28

**Authors:** Lu Li, Zhijian Qiu

**Affiliations:** 1 School of Mathematics, Southwestern University of Finance and Economics,Chengdu, Sichuan, China; Roma Tre University: Universita degli Studi Roma Tre, ITALY

## Abstract

This paper investigates the optimal robust equilibrium investment and reinsurance strategy in a model with common shock dependent claims for an ambiguity-averse insurer (AAI). Suppose that the insurance company can purchase proportional reinsurance whose reinsurance premium is calculated by the expected value principle to disperse risks. The ambiguity-averse insurer’s wealth process have two dependent classes of insurance business and the surplus can be invested in a financial market composed of one risk-free asset and one risky asset, where the risky asset’s price is characterized by the constant elasticity of variance (CEV) model. Applying the game theory framework under the mean-variance criterion, the optimal investment reinsurance problem are derived. By adopting stochastic control theory and solving the corresponding extended Hamilton-Jacobi-Bellman (HJB) equations, we obtain the robust optimal investment-reinsurance strategy and the corresponding equilibrium value function. Furthermore, some numerical examples are provided to illustrate the effects of model parameters on the optimal investment and reinsurance strategy.

## 1 Introduction

The investment and reinsurance activities of insurance companies are crucial components in their overall risk management and capital optimization strategies. Investing in financial markets allows insurers to generate risk-adjusted returns that can bolster their underwriting capabilities and solvency positions. Conversely, reinsurance facilitates risk diversification by transferring portions of an insurer’s risk exposures to reinsurance providers. In recent years, the problem of optimizing investment and reinsurance decisions has been a topic of growing academic interest. This problem involves determining the optimal allocation of an insurer’s assets, as well as the optimal level of its reinsurance program, so that the insurer’s long-term financial performance can be maximized. The existing academic literatures have employed techniques from fields such as actuarial science, financial economics, and operations research to model and solve this complex optimization problem.The researches are studied under assumptions about insurers’ objectives, constraints, and the underlying uncertainty in financial markets and insurance claim processes. Liang and Yuen [1], Yuen [2], Zhang and Siu [3] and Li [4] discussed the problem for maximizing the expected utility. Promislow and Young [5], Schmidli [6], Cao and Zeng [7] Han et al. [8] studied the optimal investment and reinsurance strategies of an insurance company with minimizing ruin probability of the risk process.

Besides, much literature has studied this topic under the mean-variance criterion which takes into account not only the risk but also the returns. Compared with the expected utility maximization criterion and the ruin probability minimization criterion, the mean-variance criterion can make the insurance companies reduce the risk as much as possible, and it has become a popular decision criterion in financial theory because of its rationality and practicability. The study of the mean-variance criterion can be traced back to the literature [9]. Thereafter, the optimization problem of the mean-variance criterion in finance and insurance has been extensively studied. It is well known that the mean-variance criterion problem is time-inconsistent due to the lack of iterative expectation attributes, so the Bellman optimality principle is not available. Generally, there are two ways to solve the time-inconsistent problem. One of the methods is to seek a precommitment strategy. For example, Li and Ng [10] firstly derived the precommitment mean-variance portfolio and turned the time-inconsistent problem into a stochastic linear quadratic (LQ) control problem by an embedding technique in the discrete-time case. After Li and Ng [10] introduced the embedding technique, Zhou et al. [11] also used the similar method to deal with the continuous-time problem. Another method is to derive equilibrium strategy within a game theoretic framework pioneered by Strotz [12] and Goldman [13]. Basak and Chabakauri [14] explored the mean-variance portfolio problem within the context of the Wiener-driven framework, ultimately deriving a time consistent strategy. For further details, refer to the works of Björk and Murgoci [15], [16] and Björk [17].

Although many scholars have investigated the time-consistent optimal investment-reinsurance strategy under the mean-variance criterion, there are still two aspects worth discussing. On the one hand, the model uncertainty can be considered in the investment–reinsurance problem. It is widely known that the AAI has faced challenges in precisely estimating the expected surpluses. Therefore, the AAI finds a way to deal with the uncertainty. Anderson [18] first proposed that robust control is the optimal control in the worst-case scenario of alternative models. Maenhout [19] studied the influence of ambiguity on intertemporal portfolio selection by introducing robust decision-making. Maenhout [19] investigated the effect of ambiguity on the intertemporal portfolio choice in a setting with constant investment opportunities and in a setting with a mean-reverting equity risk premium, respectively. Some articles are inspired by Maenhout [19] to derive the robust optimal investment strategy. Yi et al. [20] studied robust optimal strategy of the AAI for maximizing the expected terminal utility, and discussed optimal strategies under mean–variance criterion. Pun [21] proposed a framework to deal with model uncertainty under mean–variance criterion. Zhang et al. [22] introduced defaultable risks and jumped into the robust problem. On the other hand, insurance businesses often exhibit some degree of interdependence. A prime example is the COVID-19 pandemic (or other natural disasters like hurricanes or earthquakes), which can give rise to various types of insurance claims. Some researches have proposed that these cumulative claims may be interrelated through a common shock, which can capture the impact of diverse insurance claims stemming from such natural catastrophes. Yuan et al. [2] studied the investment-reinsurance problem in the case that the claim quantity processes were associated with a common shock. Zhang et al. [23] considered optimal excess-of-loss reinsurance and investment problem with thinning dependent risks.

Inspired by the previous research, and recognizing the potential deviation between estimation models and real-world market conditions, this study introduces model uncertainty and assumes that insurance companies exhibit a degree of aversion. Furthermore, we establish a conditional dependence risk model, where common shocks capture the dependency between risks. The insurance company (AAI) can invest in both risk-free and risky assets, with the risky asset characterized by the Constant Elasticity of Variance (CEV) model. Within this framework, we consider the robust optimal investment and reinsurance problem under the common shock dependent claims and the CEV model. Applying the stochastic control theory in the game-theoretic framework, we obtain the extended HJB equation. Then, we derive both the explicit expression of robust investment-reinsurance strategy and the corresponding equilibrium value function. Furthermore, there are three special cases of our model, which show that our model and results extend some ones in the existing literature. Finally, the economic significance of the research results is expounded. The main contributions of this paper are as follows: (1) We consider a general financial market, which includes one risk-free asset and one risky asset. The price process of the risky asset is governed by the CEV model. (2) We study the time-consistent investment problem under the mean-variance criterion and the corresponding equilibrium value function are obtained, which are different from Bi et al. [24] and Bi and Chen [25] who considered risk common shocks but did not consider time-consistency. (3) Compared with Pun [21], two insurance businesses with common shocks are studied and two robust reinsurance strategies which are influenced by some common factor are analyzed, thus making the discussion more realistic and complicated. (4) The influences of model parameters on the optimal time-consistent investment strategy are systematically examined through numerical experiments.

The paper is organized as follows. We describe the model structure and formulate the stochastic control problem in Sect 2. In Sect 3, by solving the extended HJB equation, we derive the robust equilibrium investment and reinsurance strategy and the corresponding equilibrium value functions. Numerical sensitivity analysis and economic explanation are illustrated in Sect 4. Sect 5 summarizes this paper.

## 2 Model settings

In this paper, we consider a probability space  ( *Ω* , *F* , *F* , *ℙ* )  equipped with a right continuous and *ℙ*-complete filtration $F:={\left\{F_{t}\right\}}_{t\in [0,T]}$, where ${\left\{F_{t}\right\}}_{t\in [0,T]}$ represents the information of the market available up to time *t*.  [ 0 , *T* ]  represents the investment period of insurance company and *T* is a fixed time horizon. *ℙ* is the reference measure. All stochastic processes below are well-defined and adapted in the filtered probability space $\left(\Omega ,F,{\left\{F_{t}\right\}}_{t\in [0,T]},\mathbb{P}\right)$. In addition, we suppose that there are no transaction costs or taxes in the financial and insurance market.

### 2.1 Dynamics of the surplus processes

Suppose an insurer has an insurance portfolio business, which is composed of two different insurance business, such as medical insurance and death insurance, the claims arrival in these lines are modeled as dependent Poisson processes with common shocks. The insurer’s reserve process is


$$\begin{array}{ccc} \hat{R}(t)=x_{0}+ct-\{\overset{N_{1}(t)+N(t)}{\underset{i=1}{\sum }}L_{i}+\overset{N_{2}(t)+N(t)}{\underset{i=1}{\sum }}Y_{i}\} & & \end{array}$$ (1)

where $x_{0}$ is a deterministic initial reserve, *c* represents the premium rate. $L_{i},i$ = 1, 2,..., is the claim size from the first-class claim and assume sequence $\left\{L_{i},i\geq 1\right\}$ are independent with common distribution $F_{L}$(·), $\mu_{L}=E(L_{i})$, and $\mu_{L}^{\prime }=E(L_{i}^{2})$. $Y_{i},i$ = 1, 2,..., is the amount of the second-class claim and assume sequence $\left\{Y_{i},i\geq 1\right\}$ are independent with common distribution $F_{Y}$(·), $\mu_{Y}=E(Y_{i})$, and $\mu_{Y}^{\prime }=E(Y_{i}^{2})$. Then $S_{1}(t):=\sum_{i=1}^{N_{1}(t)+N(t)}L_{i}$ shows the claim amount of the first-class within  [ 0 , *t* ]  and $S_{2}(t):=\sum_{i=1}^{N_{2}(t)+N(t)}Y_{i}$ is the claim amount of the second-class within  [ 0 , *t* ] , where ${\left\{N_{1}(t)\;+\;N(t)\right\}}_{t\geq 0}$ and ${\left\{N_{2}(t)\;+\;N(t)\right\}}_{t\geq 0}$ represent the number of claims for the first and second categories of insurance business up to time *t*, respectively. In addition, ${\left\{N_{1}(t)\right\}}_{t\geq 0}$, ${\left\{N_{2}(t)\right\}}_{t\geq 0}$ and ${\left\{N(t)\right\}}_{t\geq 0}$ are independent Poisson processes with intensity $\lambda_{1}>0$, $\lambda_{2}>0$ and *λ* > 0, respectively. Obviously, the dependence of the two types of business is controlled by the common impact caused by ${\left\{N(t)\right\}}_{t\geq 0}$. The larger *λ* means the higher degree of dependence of the two insurance businesses. In addition, assume that the premium rate of the insurance company is calculated based on the expected value principle:


$$c^{\prime }=(1+\theta_{1})(\lambda_{1}+\lambda )\mu_{L}+(1+\theta_{2})(\lambda_{2}+\lambda )\mu_{Y}$$

where $\theta_{1}$ and $\theta_{2}$ are the insurer’s positive safety loads for the first and second class of claims. Besides, to be protected from potential large claims, we suppose insurers can govern their insurance risks by buying proportional reinsurance, but they can not get new business. Let $q_{1}(t)(\in (0,1])$ and $q_{2}(t)(\in (0,1])$ be the reinsurance retention levels for the first class claims and the second class claims at time *t*, respectively. When a claim occurs, the insurer pays $q_{1}(t)L_{i}$ (or $q_{2}(t)Y_{i}$) and the reinsurer pays $(1-q_{1}(t))L_{i}$ (or $(1-q_{2}(t))Y_{i}$). The reinsurance premium is given by


$$c^{\prime \prime }=(1-q_{1}(t))(1+\eta_{1})(\lambda_{1}+\lambda )\mu_{L}+(1-q_{2}(t))(1+\eta_{2})(\lambda_{2}+\lambda )\mu_{Y}$$

$\eta_{1}$ and $\eta_{2}$ are positive safety loads for the first and second types of claims. Note that for the insurer, $q_{1}(t)(\in (0,1])$ and $q_{2}(t)(\in (0,1])$ correspond to a reinsurance cover and $q_{1}(t)>1$ (or $q_{2}(t)>1$) would mean that the company can take an extra insurance business from other companies (i.e., act as a reinsurer for other cedents). In general, suppose $\eta_{i}>\theta_{i}$, *i* = 1 , 2 and $\eta_{1}$ and $\eta_{2}$ are positive safety loads for the insurer. After reinsurance, the insurer’s premium becomes


$$\begin{array}{ccc} \begin{array}{ccc} C: & =c^{\prime }-c^{\prime \prime } & \\ & =[(1+\eta_{1})q_{1}(t)+\theta_{1}-\eta_{1}](\lambda_{1}+\lambda )\mu_{L}+[(1+\eta_{2})q_{2}(t)+\theta_{2}-\eta_{2}](\lambda_{2}+\lambda )\mu_{Y}, \end{array} & & \end{array}$$ (2)

the reserve process ${\left\{\hat{R}(t)\right\}}_{t\geq 0}$ evolves as


$$\begin{array}{ccc} d\hat{R}(t)=Cdt-q_{1}(t)dS_{1}(t)-q_{2}(t)dS_{2}(t). & & \end{array}$$ (3)

Similar to Liang [1] and Browne [26], for the convenience of calculation, we assume that the composite Poisson process $S_{1}(t)$ and $S_{2}(t)$ can be approximated by the following geometric Brownian motion:


$$dS_{1}(t)=a_{1}dt-\sigma_{1}dB_{1}(t),$$

with $a_{1}=(\lambda_{1}+\lambda )E(L_{i})$, and $\sigma_{1}^{2}=(\lambda_{1}+\lambda )E(L_{i}^{2})$. In the same way,


$$dS_{2}(t)=a_{2}dt-\sigma_{2}dB_{2}(t),$$

with $a_{2}=(\lambda_{2}+\lambda )E(Y_{i})$, and $\sigma_{2}^{2}=(\lambda_{2}+\lambda )E(Y_{i}^{2})$. Here $B_{1}(t)$ and $B_{2}(t)$ are standard Brownian motions. Then the reserve process can be written as:


$$\begin{array}{ccc} \begin{array}{ccc} d\hat{R}(t) & =[a_{1}(\eta_{1}q_{1}(t)+\theta_{1}-\eta_{1})+a_{2}(\eta_{2}q_{2}(t)+\theta_{2}-\eta_{2})]dt & \\ & \;+\sigma_{1}q_{1}(t)dB_{1}(t)+\sigma_{2}q_{2}(t)dB_{2}(t). \end{array} & & \end{array}$$ (4)

Referring to Browne [26], Liang and Yuen [27], the correlation coefficient of $B_{1}(t)$ and $B_{2}(t)$ is denoted as:


$$\rho =\frac{\lambda E(L_{i})E(Y_{i})}{\sqrt{\left(\lambda_{1}+\lambda )E(L_{i}^{2})(\lambda_{2}+\lambda )E(Y_{i}^{2}\right)}}=\frac{\lambda \mu_{L}\mu_{Y}}{\sigma_{1}\sigma_{2}},$$

with $E(B_{1}(t)B_{2}(t))=\rho t$. Then the reserve process is rewritten as:


$$\begin{array}{ccc} \begin{array}{ccc} d\hat{R}(t) & =[(\theta_{1}-\eta_{1})a_{1}+(\theta_{2}-\eta_{2})a_{2}+a_{1}\eta_{1}q_{1}(t)+a_{2}\eta_{2}q_{2}(t)]dt & \\ & \;+\sqrt{\sigma_{1}^{2}q_{1}^{2}(t)+\sigma_{2}^{2}q_{2}^{2}(t)+2q_{1}(t)q_{2}(t)\lambda \mu_{L}\mu_{Y}}dW_{0}(t), \end{array} & & \end{array}$$ (5)

$W_{0}(t)$ is a standard Brownian motion.

### 2.2 Financial market

Suppose the insurer can invest in one risk-free asset and one risky asset. The price process of the risk-free asset is


$$\begin{array}{ccc} \{\begin{array}{c} dS_{0}(t)=rS_{0}(t)dt,t\in [0,T],\;\\ S_{0}(0)=1,\; \end{array} & & \end{array}$$ (6)

in which *r* ( > 0 )  represents the constant risk-free interest rate. The price process of the risky asset is presented as follows:


$$\begin{array}{ccc} \{\begin{array}{c} dS(t)=S(t)[\alpha dt+\sigma S^{\beta }(t)dW(t)],t\in [0,T],\;\\ S(0)=s_{0},\; \end{array} & & \end{array}$$ (7)

where *α* ( > *r* )  is the appreciation rate, *σ* is the volatility coefficient and *β* is the elasticity parameter which satisfies the general condition *β* ≥ 0. *W *(*t*) is a standard ${\left\{F_{t}\right\}}_{t\geq 0}$-adapted Brownian motion which is independent of $W_{0}(t)$. Suppose *r*, *α* and *σ* are given. *X*(*t*) represents the insurer’s wealth at time *t* and *π* ( *t* )  represent the amount invested in the risky asset, then *X* ( *t* ) - *π* ( *t* )  is the investment amount of the risk-free asset. Define $u(t):=(q_{1}(t),q_{2}(t),\pi (t))$ as the investment-reinsurance strategy at time *t*. Let $X^{u}(t)$ represents the wealth process as we apply the strategy *u*(·). Then, we derive the following wealth dynamics depicted by a stochastic differential equation:


$$\begin{array}{ccc} \begin{array}{ccc} dX^{u}(t) & =\frac{X^{u}(t)-\pi (t)}{S_{0}(t)}dS_{0}(t)+\frac{\pi (t)}{S(t)}dS(t)+d\hat{R}(t) & \\ & =[rX^{u}(t)+(\alpha -r)\pi (t)+(\theta_{1}-\eta_{1})a_{1}+(\theta_{2}-\eta_{2})a_{2}\\ & \;+a_{1}\eta_{1}q_{1}(t)+a_{2}\eta_{2}q_{2}(t)]dt+\sigma \pi (t)S^{\beta }(t)dW(t)\\ & \;+\sqrt{\sigma_{1}^{2}q_{1}^{2}(t)+\sigma_{2}^{2}q_{2}^{2}(t)+2q_{1}(t)q_{2}(t)\lambda \mu_{L}\mu_{Y}}dW_{0}(t). \end{array} & & \end{array}$$ (8)

To simplify the formula, we suppose


$$\begin{array}{r} \zeta_{1}(t)=rX^{u}(t)+(\alpha -r)\pi (t)+(\theta_{1}-\eta_{1})a_{1}+(\theta_{2}-\eta_{2})a_{2}+a_{1}\eta_{1}q_{1}(t)+a_{2}\eta_{2}q_{2}(t) \end{array}$$

and


$$\begin{array}{r} \zeta_{2}(t)=\sqrt{\sigma_{1}^{2}q_{1}^{2}(t)+\sigma_{2}^{2}q_{2}^{2}(t)+2q_{1}(t)q_{2}(t)\lambda \mu_{L}\mu_{Y}} \end{array}$$

Then, the real wealth $X^{u}(t)$ can be described as


$$\begin{array}{ccc} dX^{u}(t)=\zeta_{1}(t)dt+\sigma \pi (t)S^{\beta }(t)dW(t)+\zeta_{2}(t)dW_{0}(t) & & \end{array}$$ (9)

### 2.3 Problem formulation

For the optimal portfolio selection problem under mean-variance criterion, the insurer’s objective varies with every state: *∀* ⁡  $(t,x,s)\in [0,T]\times \mathbb{R}\times {\mathbb{R}}^{+}$, the optimal portfolio selection problem can be represented by:


 sup ⁡ u∈Π {Et,x,s[Xu(T)]-ω2Vart,x,s[Xu(T)] }, (10)

where *ω* > 0 is the insurer’s risk-averse coefficient. The insurance company needs to formulate equilibrium time-consistent investment-reinsurance strategies.

The traditional investment-reinsurance model assumes that the insurer is ambiguity-neutral. However, in reality, insurance companies are averse to ambiguity and seek to mitigate the worst-case scenario. Following Anderson et al. [18], the accurate probability measure *ℙ* is difficult to find, so most of the insurers are ambiguity-averse, and hope to ensure that the worst-case return can achieve the expected goal, so they may take into consideration an alternative model. Then the alternative models are defined as a class of probability measures equivalent to the probability measure *ℙ* as follows:


$${\mathbb{P}}^{*}:=\{{\mathbb{P}}^{*}|{\mathbb{P}}^{*}\sim \mathbb{P}\}.$$

Define a process $\left\{\phi (t):=(\phi_{1}(t),\phi_{2}(t))|t\in [0,T]\right\}$ satisfying that:

(i) $\phi_{1}(t)$ and $\phi_{2}(t)$ are predictable with respect to $\left\{F_{t}\right\}$;

(ii) $E[e^{\frac{1}{2}\int_{0}^{T}\{{\left(\phi_{1}(t)\right)}^{2}+{\left(\phi_{2}(t)\right)}^{2}\}dt}]<\infty$.

We denote *Φ* for the space of all such processes *ϕ*. For every *ϕ* ( *t* ) ∈ *Φ*, according to Girsanov’s theorem, there exists a progressively measurable process  { *Γ* ( *t* ) | *t* ∈ [ 0 , *T* ] }  on  ( *Ω* , *F* , *F* , *ℙ* )  such that


$$\begin{array}{ccc} \begin{array}{ccc} \Gamma^{\phi }(t) & =\exp\{-\int_{0}^{t}\phi_{1}(\nu )dW_{0}(\nu )-\frac{1}{2}\int_{0}^{t}\phi_{1}^{2}(\nu )d\nu & \\ & \;-\int_{0}^{t}\phi_{2}(\nu )dW(\nu )-\frac{1}{2}\int_{0}^{t}\phi_{2}^{2}(\nu )d\nu \}. \end{array} & & \end{array}$$ (11)

Based on the *ϕ* ( *t* )  we defined before, we realize that $\Gamma^{\phi }(t)$ satisfies the following condition:


$$\begin{array}{ccc} \begin{array}{cc} d\Gamma^{\phi }(t) & =\Gamma^{\phi }(t)[-\phi (t)dW_{1}(t)],\;\Gamma^{\phi }(0)=1 \end{array} & & \end{array}$$ (12)

where $W_{1}(t)={\left(W_{0}(t),W(t)\right)}^{\prime }$. Hence, $\Gamma^{\phi }(t)$ satisfies Novikov condition, similar to Branger and Larsen [28], we notice that $\Gamma^{\phi }(t)$ is a *ℙ*-exponential martingale, then $E[\Gamma^{\phi }(t)]=1$, and the corresponding Radon-Nikodym derivative of ${\mathbb{P}}^{*}$ w.r.t *ℙ* can be defined as


$$\frac{d{\mathbb{P}}^{*}}{d\mathbb{P}}{|}_{F_{t}}=\Gamma^{\phi }(t).$$

By Girsanov’s theorem, under the alternative probability ${\mathbb{P}}^{*}$, the standard Brownian motions ${\left\{W^{{\mathbb{P}}^{*}}(t)\right\}}_{t\in [0,T]}$ and ${\left\{W_{0}^{{\mathbb{P}}^{*}}(t)\right\}}_{t\in [0,T]}$ are written as:


$$\begin{array}{ccc} \begin{array}{c} dW^{{\mathbb{P}}^{*}}(t)=dW(t)+\phi_{2}(t)dt,\;dW_{0}^{{\mathbb{P}}^{*}}(t)=dW_{0}(t)+\phi_{1}(t)dt. \end{array} & & \end{array}$$ (13)

where ${\left\{W^{{\mathbb{P}}^{*}}(t)\right\}}_{t\in [0,T]}$ and ${\left\{W_{0}^{{\mathbb{P}}^{*}}(t)\right\}}_{t\in [0,T]}$ are mutually independent. Based on the discussion above, the dynamics of the wealth process (9) under the alternative probability ${\mathbb{P}}^{*}$ become:


$$\begin{array}{ccc} \begin{array}{ccc} dX^{u}(t) & =[\zeta_{1}(t)-\zeta_{2}(t)\phi_{1}(t)-\sigma \pi (t)S^{\beta }(t)\phi_{2}(t)]dt & \\ & \;+\sigma \pi (t)S^{\beta }(t)dW^{{\mathbb{P}}^{*}}(t)+\zeta_{2}(t)dW_{0}^{{\mathbb{P}}^{*}}(t), \end{array} & & \end{array}$$ (14)

the risky asset’s price process is:


$$\begin{array}{ccc} \begin{array}{ccc} dS(t) & =S(t)[\alpha dt+\sigma S^{\beta }(t)dW^{{\mathbb{P}}^{*}}(t)-\sigma S^{\beta }(t)\phi_{2}(t)dt] & \\ & =S(t)[(\alpha -\sigma S^{\beta }(t)\phi_{2}(t))dt+\sigma S^{\beta }(t)dW^{{\mathbb{P}}^{*}}(t)]. \end{array} & & \end{array}$$ (15)

We denote the control-measure strategy by $\left(u,\phi )=(\{q_{1}(t),q_{2}(t),\pi (t)\},\{\phi (t)\}\right)$. And we formulate the following robust stochastic optimization problem based on the mean-variance criterion, we define the objective function ${\hat{J}}^{{\mathbb{P}}^{*}}(t,x,s;u,\phi )$ as follows:


$$\begin{array}{r} \begin{array}{ccc} {\hat{J}}^{{\mathbb{P}}^{*}}(t,x,s;u,\phi ) & =E_{t,x,s}^{{\mathbb{P}}^{*}}[\int_{t}^{T}(\frac{{\left(\phi_{1}(\nu )\right)}^{2}}{2\varphi_{1}(\nu )}+\frac{{\left(\phi_{2}(\nu )\right)}^{2}}{2\varphi_{2}(\nu )})d\nu & \\ & \;+E_{t,x,s}^{{\mathbb{P}}^{*}}[X^{u}(T)]-\frac{\omega }{2}Var_{t,x,s}^{{\mathbb{P}}^{*}}[X^{u}(T)]], \end{array} \end{array}$$

where $\varphi_{1}(t)$ and $\varphi_{2}(t)$ capture the AAI’s ambiguity aversions and they are nonnegative, namely, the larger $\varphi_{1}(t)$ and $\varphi_{2}(t)$, the more ambiguity-averse the AAI. Furthermore, the deviations from the reference measure *ℙ* are dependent on the relative entropy. The relative entropy is defined as the expectation under alternative measure of the log Radon-Nikodym derivative defined in Eq (12). From Ito’s Lemma we get that


dln ⁡ Λϕ(t)=-ϕ1(t)dW0(t)-12ϕ12(t)dt-ϕ2(t)dW(t)-12ϕ22(t)dt.

The deviation between the reference measure *ℙ* and the alternative measure ${\mathbb{P}}^{*}$ is captured by the relative entropy. In Appendix I of S1 Appendix, it is shown that the increase in the relative entropy from *t* to *t* + *δ* equals


$$[\frac{1}{2}({\left(\phi_{1}(t)\right)}^{2}+{\left(\phi_{2}(t)\right)}^{2})]dt.$$

When measure ${\mathbb{P}}^{*}$ deviates from *ℙ*, a positive penalty will be generated. Inspired by Maenhout [19], we suppose insurance companies seek a robust optimal control under the worst-case scenario among ${\mathbb{P}}^{*}$ for every  ( *t* , *x* , *s* ) :


 sup ⁡ u∈ΠJ(t,x,s;u,ϕ)= sup ⁡ u∈Π inf ⁡ ϕ∈ΦJ^ℙ*(t,x,s;u,ϕ), (16)

where J(t,x,s;u,ϕ)= inf ⁡ ϕ∈ΦJ^ℙ*(t,x,s;u,ϕ), we give the following definition:

**Definition 1 (Admissible Control-measure Strategy).** A control-measure strategy $\left(u,\phi )=(\{q_{1}(t),q_{2}(t),\pi (t)\},\{\phi (t)\}\right)$ is said to be admissible if it satisfies: (1) $q_{i}(t)\geq 0,i=1,2$, *t* ∈ [ 0 , *T* ]  and $E_{t,x,s}^{{\mathbb{P}}^{*}}\int_{0}^{T}[{\left(q_{1}(t)\right)}^{2}+{\left(q_{2}(t)\right)}^{2}+{\left(\pi (t)\right)}^{2}]dt<\infty$, where $E_{t,x,s}^{{\mathbb{P}}^{*}}$[·] = $E^{{\mathbb{P}}^{*}}$[·$|X^{u}(t)=x$]; (2) *u*(*t*) is ${\left\{F_{t}\right\}}_{t\in [0,T]}$-progressively measurable; (3) for all $(t,x,s)\in [0,T]\times \mathbb{R}\times {\mathbb{R}}^{+}$, Eq (8) has a unique strong solution; (4) *ϕ* ( *t* )  satisfies the Novikov condition $E[e^{\frac{1}{2}\int_{0}^{T}\{{\left(\phi_{1}(t)\right)}^{2}+{\left(\phi_{2}(t)\right)}^{2}\}dt}]<\infty .$

We denote *Π* × *Φ* as the admissible control-measure strategy set.

**Definition 2.** Consider an admissible control-measure strategy ($u^{*}$, $\phi^{*}$), which can be informally viewed as a candidate equilibrium strategy. For any fixed point $(t,x,s)\in [0,T]\;\times \;\mathbb{R}\;\times \;{\mathbb{R}}^{+}$ and *ξ* ( > 0 ) , We define


$$\begin{array}{r} u_{\xi }=\{\begin{array}{c} u^{\prime }(t,x,s),\;\;(t,x,s)\in [t,t+\xi ]\times \mathbb{R}\times {\mathbb{R}}^{+},\;\\ u^{*}(t,x,s),\;\;(t,x,s)\in [t+\xi ,T]\times \mathbb{R}\times {\mathbb{R}}^{+},\;\;\; \end{array} \end{array}$$

and


$$\begin{array}{r} \phi_{\xi }=\{\begin{array}{c} \phi^{\prime }(t,x,s),\;\;(t,x,s)\in [t,t+\xi ]\times \mathbb{R}\times {\mathbb{R}}^{+},\;\\ \phi^{*}(t,x,s),\;\;(t,x,s)\in [t+\xi ,T]\times \mathbb{R}\times {\mathbb{R}}^{+}.\;\;\; \end{array} \end{array}$$

If (i) for all $(u^{\prime },\phi^{\prime })\in \Pi \times \Phi$,


lim ⁡ ξ↓0 inf ⁡ J(t,x,s;uξ,ϕξ)-J(t,x,s;uξ,ϕ*)ξ≥0,

(ii) for all *u* ∈ *Π*,


lim ⁡ ξ↓0 inf ⁡ J(t,x,s;uξ,ϕ*)-J(t,x,s;u*,ϕ*)ξ≤0,

then $\left(u^{*},\phi^{*}\right)$ is an equilibrium control-measure strategy and equilibrium value function $J(t,x,s;u^{*},\phi^{*}$ is given by


$$J(t,x,s;u^{*},\phi^{*})=E_{t,x,s}^{{\mathbb{P}}^{*}}[X^{u^{*}}(T)]-\frac{\omega }{2}Var_{t,x,s}^{{\mathbb{P}}^{*}}[X^{u^{*}}(T)].$$

where *ω* > 0 is the insurer’s risk-averse coefficient and $J(t,x,s;u^{*},\phi^{*})$ is evaluated with ${\mathbb{P}}^{*}$.

According to Definition 2, the equilibrium strategy above is time-consistent. We aim to seek an equilibrium strategy ($u^{*}$, $\phi^{*}$) and the corresponding equilibrium value function. To give the extended HJB equation and Verification Theorem conveniently, we define a variational operator.

Denote that $C^{1,2,2}([0,T]\times \mathbb{R}\times {\mathbb{R}}^{+})$ is the space of the function *Ψ* ( *t* , *x* , *s* )  such that *Ψ* ( *t* , *x* , *s* )  and its derivatives $\Psi_{t}(t,x,s)$, $\Psi_{x}(t,x,s)$, $\Psi_{xx}(t,x,s)$, $\Psi_{s}(t,x,s)$,$\Psi_{ss}(t,x,s)$ and $\Psi_{xs}(t,x,s)$ are continuous on $[0,T]\times \mathbb{R}\times {\mathbb{R}}^{+}$. Define operator $L^{u,\phi }$ before giving the verification theorem: For $\forall (t,x,s)\in [0,T]\times \mathbb{R}\times {\mathbb{R}}^{+}$, $\forall \Psi (t,x,s)\in C^{1,2,2}([0,T]\times \mathbb{R}\times {\mathbb{R}}^{+})$, the variational operator corresponding to the alternative measure ${\mathbb{P}}^{*}$ is defined as follows:


$$\begin{array}{ccc} \begin{array}{ccc} L^{u,\phi }\Psi (t,x,s) & =\Psi_{t}(t,x,s)+[\zeta_{1}(t)-\sigma \pi (t)s^{\beta }\phi_{2}(t)-\zeta_{2}(t)\phi_{1}(t)]\Psi_{x}(t,x,s) & \\ & \;+(\alpha s-\sigma s^{\beta +1}\phi_{2}(t))\Psi_{s}(t,x,s)+\frac{1}{2}(\sigma^{2}\pi^{2}(t)s^{2\beta }+\zeta_{2}^{2}(t))\Psi_{xx}(t,x,s)\\ & \;+\frac{1}{2}\sigma^{2}s^{2\beta +2}\Psi_{ss}+\sigma^{2}\pi (t)s^{2\beta +1}\Psi_{xs}(t,x,s). \end{array} & & \end{array}$$ (17)

Before finding the robust equilibrium strategy, the following theorem gives the verifications for the extended HJB equation corresponding to the problem (16).

**Theorem 3 (Verification Theorem).** For the problem (16), we suppose that there exists two real-valued functions *V* ( *t* , *x* , *s* ) , *g* ( *t* , *x* , *s* ) $\in C^{1,2,2}([0,T]\;\times \;\mathbb{R}\;\times \;{\mathbb{R}}^{+})$ satisfying the following extended HJB equation:


sup ⁡ u∈Π inf ⁡ ϕ∈Φ {Lu,ϕV(t,x,s)-Lu,ϕω2g2(t,x,s)+ωg(t,x,s)Lu,ϕg(t,x,s)+ϕ12(t)2φ1(t)+ϕ22(t)2φ2(t) }=0, (18)

$$\begin{array}{ccc} L^{u^{*},\phi^{*}}g(t,x,s)=0,V(T,x,s)=x,g(T,x,s)=x. & & \end{array}$$ (19)

and


(u*,ϕ*):=arg ⁡ sup ⁡ u∈Π inf ⁡ ϕ∈Φ {Lu,ϕV(t,x,s)-Lu,ϕω2g2(t,x,s)+ωg(t,x,s)Lu,ϕg(t,x,s)+ϕ12(t)2φ1(t)+ϕ22(t)2φ2(t) }. (20)

then *W* ( *t* , *x* , *s* ) = *V* ( *t* , *x* , *s* ) , $E^{{\mathbb{P}}^{*}}[X^{u^{*}}(T)]=g(t,x,s)$ and $\left(u^{*},\phi^{*}\right)$ is the robust time-consistent control strategy. The proof is similar to Theorem 4.1 of Björk [15].

## 3 The solution to the optimization problem

In this section, we derive the explicit solution to the robust equilibrium investment reinsurance strategy and the corresponding robust equilibrium value function. According to Maenhout [19], to make the problem (16) tractable and ensure that the penalty in problem (16) is reasonable, some restrictions must be imposed on the ambiguity-aversion parameter. We suppose the ambiguity-aversion coefficients $\varphi_{1}(t)=k_{1}\geq 0$ and $\varphi_{2}(t)=k_{2}\geq 0$ (cf. Maenhout [19]). Then according to the variational operator (17), Eq (18) can be rewritten as:


 sup ⁡ u∈Π inf ⁡ ϕ∈Φ {Vt+[ζ1(t)-ζ2(t)ϕ1(t)-σπ(t)sβϕ2(t)]Vx+12(σ2π2(t)s2β+ζ22(t))(Vxx-ωgx2)+12σ2s2β+2(Vss-ωgs2)+(αs-σsβ+1ϕ2(t))Vs+σ2π(t)s2β+1(Vxs-ωgsgx)+ϕ12(t)2k1+ϕ22(t)2k2 }=0. (21)

where the terminal value conditions are given by *V* ( *T* , *x* , *s* ) = *x* and *g* ( *T* , *x* , *s* ) = *x* . 

To guarantee the insurance retention $q_{1}(t)$ and $q_{2}(t)$ are non-negative, we give the following lemma.

**Lemma 4.** The parameters $\lambda ,\lambda_{1},\lambda_{2},\mu_{L},\mu_{Y},\mu_{L}^{\prime }$ and $\mu_{Y}^{\prime }$ are given in Sect 2 above and satisfy the following inequalities:


$$\sigma_{1}^{2}\sigma_{2}^{2}>\lambda^{2}\mu_{L}^{2}\mu_{Y}^{2},\frac{\lambda \mu_{L}\mu_{Y}}{\sigma_{2}^{2}}\frac{a_{2}}{a_{1}}<1<\frac{\sigma_{1}^{2}}{\lambda \mu_{L}\mu_{Y}}\frac{a_{2}}{a_{1}}$$

Proof. See Appendix II of S1 Appendix.

Considering $q_{1}(t)$ and $q_{2}(t)$ are non-negative, according to Lemma 4, the following cases need to be discussed.

Case 1: For $\eta_{1}<\frac{\lambda \mu_{L}\mu_{Y}}{(\lambda +\lambda_{2})\mu_{Y}^{\prime }}\frac{(\lambda +\lambda_{2})\mu_{Y}}{(\lambda +\lambda_{1})\mu_{L}}\eta_{2}$, we have $m_{1}\leq 0$ and $m_{2}>0$;

Case 2: For $\frac{\lambda \mu_{L}\mu_{Y}}{(\lambda +\lambda_{2})\mu_{Y}^{\prime }}\frac{(\lambda +\lambda_{2})\mu_{Y}}{(\lambda +\lambda_{1})\mu_{L}}\eta_{2}<\eta_{1}<\frac{(\lambda +\lambda_{1})\mu_{L}^{\prime }}{\lambda \mu_{L}\mu_{Y}}\frac{(\lambda +\lambda_{2})\mu_{Y}}{(\lambda +\lambda_{1})\mu_{L}}\eta_{2}$, we have $m_{1}>0$ and $m_{2}>0$;

Case 3: For $\eta_{1}\geq \frac{(\lambda +\lambda_{1})\mu_{L}^{\prime }}{\lambda \mu_{L}\mu_{Y}}\frac{(\lambda +\lambda_{2})\mu_{Y}}{(\lambda +\lambda_{1})\mu_{L}}\eta_{2}$, we have $m_{1}>0$ and $m_{2}\leq 0$.

Here $m_{1}=\frac{a_{1}\eta_{1}\sigma_{2}^{2}-a_{2}\eta_{2}\lambda \mu_{L}\mu_{Y}}{\sigma_{1}^{2}\sigma_{2}^{2}-\lambda^{2}\mu_{L}^{2}\mu_{Y}^{2}}$ and $m_{2}=\frac{a_{2}\eta_{2}\sigma_{1}^{2}-a_{1}\eta_{1}\lambda \mu_{L}\mu_{Y}}{\sigma_{1}^{2}\sigma_{2}^{2}-\lambda^{2}\mu_{L}^{2}\mu_{Y}^{2}}$. We only detail analyze case 2 in the following theorem. The other two cases can be similarly deduced.

**Theorem 5.** The robust equilibrium strategy of the mean-variance problem (16) under case 2 are given by


$$\begin{array}{ccc} \begin{array}{ccc} \pi^{*}(t) & =\frac{\left(\alpha -r)+2\sigma^{2}\beta (\omega Q^{*}(t)+k_{2}F^{*}(t)\right)}{\sigma^{2}s^{2\beta }(\omega +k_{2})e^{r(T-t)}}, & \\ q_{1}^{*}(t) & =\frac{m_{1}}{(\omega +k_{1})e^{r(T-t)}},\\ q_{2}^{*}(t) & =\frac{m_{2}}{(\omega +k_{1})e^{r(T-t)}}, \end{array} & & \end{array}$$ (22)

where $m_{1}>0$ and $m_{2}>0$. The corresponding robust equilibrium value function is


$$\begin{array}{ccc} \begin{array}{ccc} V(t,x,s)= & e^{r(T-t)}x+F(t)s^{-2\beta }+\int_{t}^{T}\{\frac{-1}{2(\omega +k_{1})}(\sigma_{1}^{2}+\sigma_{2}^{2}+2m_{1}m_{2}\lambda \mu_{L}\mu_{Y}) & \\ & +(2\beta^{2}+\beta )\sigma^{2}F(\nu +e^{r(T-\nu )}(\theta_{1}-\eta_{1})a_{1}+e^{r(T-\nu )}(\theta_{2}-\eta_{2})a_{2}\\ & +\frac{1}{\omega +k_{1}}(a_{1}\eta_{1}+a_{2}\eta_{2})\}d\nu , \end{array} & & \end{array}$$ (23)

*F*(*t*) and *Q*(*t*) are determined by the following ordinary differential equations:


$$\begin{array}{ccc} \{\begin{array}{c} F^{\prime }(t)+F^{2}(t)(-2k_{2}\sigma^{2}\beta^{2}+\frac{2k_{2}^{2}\sigma^{2}\beta^{2}}{\omega +k_{2}})+F(t)(\frac{4\omega \beta^{2}\sigma^{2}Q(t)k_{2}+2(\alpha -r)k_{2}\beta }{\omega +k_{2}}-2\alpha \beta )\;\\ \;+\frac{{\left(\alpha -r\right)}^{2}}{2\sigma^{2}(\omega +k_{2})}+\frac{2\omega^{2}\beta^{2}\sigma^{2}Q{\left(t\right)}^{2}}{\omega +k_{2}}+\frac{2(\alpha -r)\omega \beta Q(t)}{\omega +k_{2}}-2\omega \sigma^{2}\beta^{2}Q{\left(t\right)}^{2}=0,\;\\ F(T)=0,\;\\ Q^{\prime }(t)+Q^{2}(t)\frac{4\omega k_{2}^{2}\sigma^{2}\beta^{2}}{{\left(\omega +k_{2}\right)}^{2}}+Q(t)(\frac{\left(2\omega^{2}\beta \sigma^{2}+2k_{2}^{2}\sigma^{2}\beta )(\alpha -r+2k_{2}\sigma^{2}\beta F(t)\right)}{{\left(\omega +k_{2}\right)}^{2}\sigma^{2}}-2\alpha \beta \;\\ \;-4\sigma^{2}k_{2}\beta^{2}F(t))+\frac{\omega {\left(\alpha -r+2k_{2}\sigma^{2}\beta F(t)\right)}^{2}}{{\left(\omega +k_{2}\right)}^{2}\sigma^{2}}=0,\;\\ Q(T)=0.\; \end{array} & & \end{array}$$ (24)

*Proof *. On the basis of the terminal condition of *V* ( *t* , *x* , *s* )  and *g* ( *t* , *x* , *s* ) , we infer the following form:


$$\begin{array}{ccc} V(t,x,s) & =H(t)x+F(t)s^{-2\beta }+G(t), & \end{array}$$ (25)

$$\begin{array}{cc} g(t,x,s) & =P(t)x+Q(t)s^{-2\beta }+R(t), \end{array}$$ (26)

where *H* ( *T* ) = *P* ( *T* ) = 1 and *F* ( *T* ) = *Q* ( *T* ) = 0. We calculate the derivatives of *V* ( *t* , *x* , *s* )  and *g* ( *t* , *x* , *s* ) , and then substitute them into Eq (21) Hence we get


$$\begin{array}{ccc} \begin{array}{ccc} \phi_{1}^{*}(t) & =k_{1}\zeta_{2}(t)H(t), & \\ \phi_{2}^{*}(t) & =k_{2}(\sigma \pi (t)s^{\beta }H(t)-2\sigma \beta F(t)s^{-\beta }). \end{array} & & \end{array}$$ (27)

By the first-order condition we get


$$\begin{array}{ccc} \begin{array}{ccc} \pi^{*}(t) & =\frac{\left(\alpha -r)H(t)+2\sigma^{2}\beta (\omega P(t)Q(t)+k_{2}H(t)F(t)\right)}{\sigma^{2}s^{2\beta }(\omega P^{2}(t)+k_{2}H^{2}(t))}, & \\ q_{1}^{*}(t) & =m_{1}\frac{H(t)}{\omega P^{2}(t)+k_{1}H^{2}(t)},\\ q_{2}^{*}(t) & =m_{2}\frac{H(t)}{\omega P^{2}(t)+k_{1}H^{2}(t)}, \end{array} & & \end{array}$$ (28)

where $m_{1}=\frac{a_{1}\eta_{1}\sigma_{2}^{2}-a_{2}\eta_{2}\lambda \mu_{L}\mu_{Y}}{\sigma_{1}^{2}\sigma_{2}^{2}-\lambda^{2}\mu_{L}^{2}\mu_{Y}^{2}}$ and $m_{2}=\frac{a_{2}\eta_{2}\sigma_{1}^{2}-a_{1}\eta_{1}\lambda \mu_{L}\mu_{Y}}{\sigma_{1}^{2}\sigma_{2}^{2}-\lambda^{2}\mu_{L}^{2}\mu_{Y}^{2}}$. Taking the above conditions, we can derive differential equations of H(t), F(t), Q(t) and P(t). Through the separation of variable *x* and $s^{-2\beta }$, we can get the more simplified differential equations. Considering the boundary conditions *H* ( *T* ) = 1 , *P* ( *T* ) = 1 , *F* ( *T* ) = 0 , *Q* ( *T* ) = 0, then we have


$$\begin{array}{ccc} \begin{array}{c} H(t)=P(t)=e^{r(T-t)}, \end{array} & & \end{array}$$ (29)

*F*(*t*) and *Q*(*t*) are decided by the differential equation which has a unique solution (cf. [29]), then we obtain the expression of *G*(*t*) and *R*(*t*):


$$\begin{array}{ccc} \begin{array}{ccc} G(t)= & \int_{t}^{T}\{\frac{-1}{2(\omega +k_{1})}(\sigma_{1}^{2}+\sigma_{2}^{2}+2m_{1}m_{2}\lambda \mu_{L}\mu_{Y})+(2\beta^{2}+\beta )\sigma^{2}F(\nu ) & \\ & \;+e^{r(T-\nu )}(\theta_{1}-\eta_{1})a_{1}+e^{r(T-\nu )}(\theta_{2}-\eta_{2})a_{2}+\frac{1}{\omega +k_{1}}(a_{1}\eta_{1}+a_{2}\eta_{2})\}d\nu , \end{array} & & \end{array}$$ (30)

$$\begin{array}{ccc} \begin{array}{ccc} R(t)= & \int_{t}^{T}\{P(\nu )(\theta_{1}-\eta_{1})a_{1}+P(\nu )(\theta_{2}-\eta_{2})a_{2}+\frac{a_{1}\theta_{1}m_{1}+a_{2}\theta_{2}m_{2}}{\omega +k_{1}} & \\ & \;-\frac{k_{1}}{{\left(\omega +k_{1}\right)}^{2}}(\sigma_{1}^{2}m_{1}^{2}+\sigma_{2}^{2}m_{2}^{2}+2\lambda \mu_{L}\mu_{Y}m_{1}m_{2})+(2\beta^{2}+\beta )\sigma^{2}Q(\nu )\}d\nu . \end{array} & & \end{array}$$ (31)

The detailed proof is shown in Appendix III of S1 Appendix.

Furthermore, based on the reinsurance strategies, it can be inferred that they are related to $m_{1}$ and $m_{2}$. Therefore, the reinsurance strategies are discussed in more detail based on the following two cases: According to the structure of $m_{1}$ and $m_{2}$, we suppose $D_{1}=a_{1}\eta_{1}\sigma_{2}^{2}\;-\;a_{2}\eta_{2}\lambda \mu_{L}\mu_{Y},D_{2}=a_{2}\eta_{2}\sigma_{1}^{2}-a_{1}\eta_{1}\lambda \mu_{L}\mu_{Y},D_{3}=\sigma_{1}^{2}\sigma_{2}^{2}-\lambda^{2}\mu_{L}^{2}\mu_{Y}^{2}.$ Note that $D_{1},D_{2},D_{3}>0$, thus $q_{1}^{*}(t)>0,q_{2}^{*}(t)>0$.

**Remark 6.** *Note that for the insurer,* $q_{1}(t)\in [0,1]$ *and* $q_{2}(t)\in ([0,1]$ *correspond to a reinsurance cover. If* $0<q_{i}^{*}(t)<1$, *then the equilibrium reinsurance strategy is* $q_{i}^{*}(t)$ *which is given by (22); if* $q_{i}^{*}(t)<0$, *then* $q_{i}^{*}(t)=0$; *if* $q_{i}^{*}(t)>1$, *then* $q_{i}^{*}(t)=1$, *this case means that the company can take an extra insurance business from other companies (i.e., act as a reinsurer for other cedents)*.

Furthermore, we let


$$\begin{array}{ccc} t_{1}=\{\begin{array}{c} T,\;\;\;\;\;\;D_{1}\leq (\omega +k_{1})D_{3},\;\\ T-\frac{1}{r}ln\frac{D_{1}}{(\omega +k_{1})D_{3}},(\omega +k_{1})D_{3}<D_{1}<(\omega +k_{1})D_{3}e^{rT},\;\\ 0,\;\;\;\;\;\;D_{1}\geq (\omega +k_{1})D_{3}e^{rT},\;\;\; \end{array} & & \end{array}$$ (32)

$$\begin{array}{ccc} t_{2}=\{\begin{array}{c} T,\;\;\;\;\;\;D_{2}\leq (\omega +k_{1})D_{3},\;\\ T-\frac{1}{r}ln\frac{D_{2}}{(\omega +k_{1})D_{3}},(\omega +k_{1})D_{3}<D_{2}<(\omega +k_{1})D_{3}e^{rT},\;\\ 0,\;\;\;\;\;\;D_{2}\geq (\omega +k_{1})D_{3}e^{rT},\;\;\; \end{array} & & \end{array}$$ (33)

Based on (31) and (32), the relationship between the size of $D_{1}$ and $D_{2}$ will determine the relationship between the size of $t_{1}$ and $t_{2}$, and the values of $t_{1}$ and $t_{2}$ will directly affect the optimal reinsurance strategy, so we will discuss it according to the case $D_{1}\leq D_{2}$ and the case $D_{1}>D_{2}$.

**Case 1 $D_{1}\leq D_{2}$**, then $t_{1}\geq t_{2}$.

(1) When $0\leq t<t_{2}$, the optimal reinsurance proportion are given by (22).

(2) When $t\geq t_{2}$, we have $q_{2}^{*}(t)=1$, according to (21), we let

$f(q_{1},q_{2},t)=(a_{1}\eta_{1}q_{1}+a_{2}\eta_{2}q_{2})H(t)-\frac{\omega +k_{1}}{2}(\sigma_{1}^{2}q_{1}^{2}+\sigma_{2}^{2}q_{2}^{2}+2\lambda \mu_{L}\mu_{Y}q_{1}q_{2})H^{2}(t)$

So, in order to solve the Eq (21), we need to solve  inf ⁡ q1,q2{f(q1,q2,t)}. Then, substituting $q_{2}^{*}(t)=1$ into  inf ⁡ q1,q2{f(q1,q2,t)} and by the first-order conditions we get


$$\begin{array}{ccc} q_{1}^{*}(t)=\frac{a_{1}\eta_{1}-\lambda \mu_{L}\mu_{Y}(\omega +k_{1})e^{r(T-t)}}{\sigma_{1}^{2}(\omega +k_{1})e^{r(T-t)}}. & & \end{array}$$ (34)

Let $t_{1}^{\prime }=T-\frac{1}{r}ln\frac{a_{1}\eta_{1}}{\left(\omega +k_{1})(\sigma_{1}^{2}+\lambda \mu_{L}\mu_{Y}\right)}$, then for $t_{2}\leq t<t_{1}^{\prime }$, the optimal reinsurance strategies are given by


$$\begin{array}{llll} q_{1}^{*}(t) & =\frac{a_{1}\eta_{1}-\lambda \mu_{L}\mu_{Y}(\omega +k_{1})e^{r(T-t)}}{\sigma_{1}^{2}(\omega +k_{1})e^{r(T-t)}},\; & & \;\\ q_{2}^{*}(t) & =1. \end{array}$$ (35)

(3) For $t_{1}^{\prime }\leq t<T$, it is easy to see that $q_{1}^{*}(t)=1,q_{2}^{*}(t)=1.$

**Case 2 $D_{1}>D_{2}$**, then $t_{1}<t_{2}$.

(1) When $0\leq t<t_{1}$, the optimal reinsurance proportion are given by (22).

(2) When $t\geq t_{1}$, we have $q_{1}^{*}(t)=1$. Substituting $q_{1}^{*}(t)=1$ into  inf ⁡ q1,q2{f(q1,q2,t)} and by the first-order conditions we get


$$\begin{array}{ccc} q_{2}^{*}(t)=\frac{a_{2}\eta_{2}-\lambda \mu_{L}\mu_{Y}(\omega +k_{1})e^{r(T-t)}}{\sigma_{2}^{2}(\omega +k_{1})e^{r(T-t)}}. & & \end{array}$$ (36)

Let $t_{2}^{\prime }=T-\frac{1}{r}ln\frac{a_{2}\eta_{2}}{\left(\omega +k_{1})(\sigma_{2}^{2}+\lambda \mu_{L}\mu_{Y}\right)}$, then for $t_{2}\leq t<t_{1}^{\prime }$, the optimal reinsurance strategies are given by


$$\begin{array}{ccc} \begin{array}{ccc} q_{1}^{*}(t) & =1, & \\ q_{2}^{*}(t) & =\frac{a_{2}\eta_{2}-\lambda \mu_{L}\mu_{Y}(\omega +k_{1})e^{r(T-t)}}{\sigma_{2}^{2}(\omega +k_{1})e^{r(T-t)}}. \end{array} & & \end{array}$$ (37)

(3) For $t_{2}^{\prime }\leq t<T$, it is easy to see that $q_{1}^{*}(t)=1,q_{2}^{*}(t)=1.$

According to the above results, we can obtain that:

(1) When $\eta_{1}<\frac{\lambda \mu_{L}\mu_{Y}}{(\lambda +\lambda_{2})\mu_{Y}^{\prime }}\frac{(\lambda +\lambda_{2})\mu_{Y}}{(\lambda +\lambda_{1})\mu_{L}}\eta_{2}$, we derive


$$\begin{array}{ccc} \begin{array}{ccc} q_{1}^{*}(t) & =0, & \\ q_{2}^{*}(t) & =\frac{a_{2}\eta_{2}}{\sigma_{2}^{2}(\omega +k_{1})e^{r(T-t)}}. \end{array} & & \end{array}$$ (38)

(2) When $\frac{\lambda \mu_{L}\mu_{Y}}{(\lambda +\lambda_{2})\mu_{Y}^{\prime }}\frac{(\lambda +\lambda_{2})\mu_{Y}}{(\lambda +\lambda_{1})\mu_{L}}\eta_{2}<\eta_{1}<\frac{(\lambda +\lambda_{1})\mu_{L}^{\prime }}{\lambda \mu_{L}\mu_{Y}}\frac{(\lambda +\lambda_{2})\mu_{Y}}{(\lambda +\lambda_{1})\mu_{L}}\eta_{2}$, we have (i) $D_{1}\leq D_{2}$, the optimal reinsurance strategies are


$$\begin{array}{ccc} (q_{1}^{*}(t),q_{2}^{*}(t))=\{\begin{array}{c} (\frac{D_{1}}{(\omega +k_{1})e^{r(T-t)}D_{3}},\frac{D_{2}}{(\omega +k_{1})e^{r(T-t)}D_{3}}),\;0\leq t<t_{2},\;\\ (\frac{a_{1}\eta_{1}-\lambda \mu_{L}\mu_{Y}(\omega +k_{1})e^{r(T-t)}}{\sigma_{1}^{2}(\omega +k_{1})e^{r(T-t)}},1),\;\;\;t_{2}\leq t<t_{1}^{\prime },\;\\ (1,1),\;\;\;\;\;\;\;t_{1}^{\prime }\leq t<T,\;\;\; \end{array} & & \end{array}$$ (39)

(ii) $D_{1}>D_{2}$, the optimal reinsurance strategies are


$$\begin{array}{ccc} (q_{1}^{*}(t),q_{2}^{*}(t))=\{\begin{array}{c} (\frac{D_{1}}{(\omega +k_{1})e^{r(T-t)}D_{3}},\frac{D_{2}}{(\omega +k_{1})e^{r(T-t)}D_{3}}),\;0\leq t<t_{1},\;\\ (1,\frac{a_{2}\eta_{2}-\lambda \mu_{L}\mu_{Y}(\omega +k_{1})e^{r(T-t)}}{\sigma_{2}^{2}(\omega +k_{1})e^{r(T-t)}}),\;\;\;t_{1}\leq t<t_{2}^{\prime },\;\\ (1,1),\;\;\;\;\;\;\;t_{2}^{\prime }\leq t<T,\;\;\; \end{array} & & \end{array}$$ (40)

(3) When $\eta_{1}\geq \frac{(\lambda +\lambda_{1})\mu_{L}^{\prime }}{\lambda \mu_{L}\mu_{Y}}\frac{(\lambda +\lambda_{2})\mu_{Y}}{(\lambda +\lambda_{1})\mu_{L}}\eta_{2}$, we have


$$\begin{array}{ccc} \begin{array}{ccc} q_{1}^{*}(t) & =\frac{a_{1}\eta_{1}-\lambda \mu_{L}\mu_{Y}(\omega +k_{1})e^{r(T-t)}}{\sigma_{1}^{2}(\omega +k_{1})e^{r(T-t)}}, & \\ q_{2}^{*}(t) & =0. \end{array} & & \end{array}$$ (41)

Next, we are going to discuss the model in three special cases, namely the ambiguity-neutral insurer (ANI) case, the investment-only case as well as the geometric Brownian motion (GBM) case.

**Remark 7.** (ANI Case). If $k_{1}=k_{2}=0$, our optimization problem (16) for an AAI is transformed into the problem for an ANI. The ANI’s wealth process is expressed as


$$\begin{array}{ccc} \begin{array}{ccc} dX^{1}(t) & =[rX^{1}(t)+(\alpha -r)\pi_{1}(t)+(\theta_{1}-\eta_{1})a_{1}+(\theta_{2}-\eta_{2})a_{2}+a_{1}\eta_{1}q_{1}(t)+a_{2}\eta_{2}q_{2}(t) & \\ & \;-\sqrt{\sigma_{1}^{2}q_{1}^{2}(t)+\sigma_{2}^{2}q_{2}^{2}(t)+2q_{1}(t)q_{2}(t)\lambda \mu_{L}\mu_{Y}}\phi_{1}(t)\\ & \;-\sigma \pi_{1}(t)S^{\beta }(t)\phi_{2}(t)]dt+\sigma \pi_{1}(t)S^{\beta }(t)dW^{\mathbb{P}}(t)\\ & \;+\sqrt{\sigma_{1}^{2}q_{1}^{2}(t)+\sigma_{2}^{2}q_{2}^{2}(t)+2q_{1}(t)q_{2}(t)\lambda \mu_{L}\mu_{Y}}dW_{0}^{\mathbb{P}}(t), \end{array} & & \end{array}$$ (42)

and the optimization problem is


sup ⁡ u1∈Π {Et,x,sℙ[X1(T)]-ω2Vart,x,sℙ[X1(T)] }. (43)

The ANI’s optimal value function and strategy are obtained.

**Proposition 8.** For optimization problem sup ⁡ u1∈Π {Et,x,sℙ[X1(T)]-ω2Vart,x,sℙ[X1(T)] }, the value function for an ANI is


$$\begin{array}{ccc} \begin{array}{ccc} V^{1}(t,x,s) & =e^{r(T-t)}x+\{\frac{{\left(\alpha -r\right)}^{2}(r-2\alpha )}{4\alpha \beta \omega r\sigma^{2}}+\frac{{\left(\alpha -r\right)}^{3}}{2\alpha \beta \omega r\sigma^{2}}e^{2\beta r(t-T)}\}\{e^{2\alpha \beta (t-T)}-1\}s^{-2\beta } & \\ & \;+\int_{t}^{T}\{-\frac{1}{2\omega }(\sigma_{1}^{2}+\sigma_{2}^{2}+2m_{1}m_{2}\lambda \mu_{L}\mu_{Y})+(2\beta^{2}+\beta )\sigma^{2}\{\frac{{\left(\alpha -r\right)}^{2}(r-2\alpha )}{4\alpha \beta \omega r\sigma^{2}}\\ & \;+\frac{{\left(\alpha -r\right)}^{3}}{2\alpha \beta \omega r\sigma^{2}}e^{2\beta r(t-T)}\}\{e^{2\alpha \beta (t-T)}-1\}+e^{r(T-\nu )}(\nu )(\theta_{1}-\eta_{1})a_{1}\\ & \;+e^{r(T-\nu )}(\theta_{2}-\eta_{2})a_{2}+\frac{1}{\omega }(a_{1}\eta_{1}+a_{2}\eta_{2})\}d\nu , \end{array} & & \end{array}$$ (44)

the optimal strategy is


$$\begin{array}{ccc} \begin{array}{ccc} \pi^{1*}(t) & =\frac{\left(\alpha -r)-\frac{{\left(\alpha -r\right)}^{2}}{r}(e^{2\beta r(T-t)}-1\right)}{\sigma^{2}s^{2\beta }\omega e^{r(T-t)}}, & \\ q_{1}^{1*}(t) & =\frac{m_{1}}{\omega e^{r(T-t)}},\\ q_{2}^{1*}(t) & =\frac{m_{2}}{\omega e^{r(T-t)}}, \end{array} & & \end{array}$$ (45)

where $m_{1}=\frac{a_{1}\eta_{1}\sigma_{2}^{2}-a_{2}\eta_{2}\lambda \mu_{L}\mu_{Y}}{\sigma_{1}^{2}\sigma_{2}^{2}-\lambda^{2}\mu_{L}^{2}\mu_{Y}^{2}}$ and $m_{2}=\frac{a_{2}\eta_{2}\sigma_{1}^{2}-a_{1}\eta_{1}\lambda \mu_{L}\mu_{Y}}{\sigma_{1}^{2}\sigma_{2}^{2}-\lambda^{2}\mu_{L}^{2}\mu_{Y}^{2}}$.

From Proposition 8, we find that the ANI’s investment strategy of Eq (43) can reduce to the case in Li [30] with *β* = 0, that is, the risky asset follows a GBM model. However, it’s well-known that the CEV model can capture volatility clustering, which is a common feature in real markets where volatility tends to persist at high or low levels for certain periods. This is something that traditional models like GBM model cannot capture. Compared with Zeng and Li [30], we formulate a more robust optimization problem with CEV mode. Besides, from the above results, it can be seen that since the CEV model has fewer parameters than the Heston model, it may be simpler and faster in terms of parameter estimation and model calibration. Furthermore, if the common shock is not considered (i.e., *λ* = 0), then


$$q_{1}^{1*}(t)=\frac{\mu_{L}\eta_{1}}{\mu_{L}^{\prime }\omega e^{r(T-t)}},q_{2}^{1*}(t)=\frac{\mu_{Y}\eta_{2}}{\mu_{Y}^{\prime }\omega e^{r(T-t)}}.$$

The reinsurance strategy is similar to Li et al. [31], but we apply another calculation principle, which makes a difference from [31]. In addition, we can find that the value function is larger than that given in Li et al. [31] due to the increase of insurance business lines in this paper.

**Remark 9.** (No reinsurance Case). If $q_{1}^{*}(t)=q_{2}^{*}(t)=1$ in Eq (21), namely the problem (16) reduces to an investment-only problem. The wealth process becomes


$$\begin{array}{ccc} \begin{array}{ccc} dX^{2}(t) & =[rX^{2}(t)+(\alpha -r)\pi_{2}(t)+(\theta_{1}-\eta_{1})a_{1}+(\theta_{2}-\eta_{2})a_{2}+a_{1}\eta_{1}+a_{2}\eta_{2} & \\ & \;-\sqrt{\sigma_{1}^{2}+\sigma_{2}^{2}+2\lambda \mu_{L}\mu_{Y}}\phi_{2}(t)-\sigma \pi_{2}(t)S^{\beta }(t)\phi_{2}(t)]dt+\sigma \pi_{1}(t)S^{\beta }(t)dW^{\mathbb{P}*}(t)\\ & \;+\sqrt{\sigma_{1}^{2}+\sigma_{2}^{2}+2\lambda \mu_{L}\mu_{Y}}dW_{0}^{\mathbb{P}*}(t), \end{array} & & \end{array}$$ (46)

and the optimization problem is


sup ⁡ u2∈Π inf ⁡ ϕ∈Φ {Lu2,ϕV(t,x,s)-Lu2,ϕω2g2(t,x,s)+ωg(t,x,s)Lu2,ϕg(t,x,s)+ϕ12(t)2φ1(t)+ϕ22(t)2φ2(t) }=0. (47)

we get the following proposition.

**Proposition 10.** When investment-only is discussed in problem (16), the value function is expressed as


$$\begin{array}{ccc} \begin{array}{ccc} V^{2}(t,x,s) & =e^{r(T-t)}x+F_{2}(t)s^{-2\beta }+\int_{t}^{T}\{-\frac{(\omega +k_{1})e^{2r(T-\nu )}}{2}(\sigma_{1}^{2}+\sigma_{2}^{2} & \\ & \;+2m_{1}m_{2}\lambda \mu_{L}\mu_{Y})+(2\beta^{2}+\beta )\sigma^{2}F_{2}(t)+e^{r(T-\nu )}(\theta_{1}-\eta_{1})a_{1}\\ & \;+e^{r(T-\nu )}(\theta_{2}-\eta_{2})a_{2}+e^{r(T-\nu )}(a_{1}\eta_{1}+a_{2}\eta_{2})\}d\nu , \end{array} & & \end{array}$$ (48)

where $F_{2}(t)$ is determined by the following equations:


$$\begin{array}{ccc} \{\begin{array}{c} F_{2}^{\prime }(t)+F_{2}^{2}(t)(-2k_{2}\sigma^{2}\beta^{2}+\frac{2k_{2}^{2}\sigma^{2}\beta^{2}}{\omega +k_{2}})+F(t)(\frac{4\omega \beta^{2}\sigma^{2}Q_{2}(t)k_{2}+2(\alpha -r)k_{2}\beta }{\omega +k_{2}}-2\alpha \beta )\;\\ \;+\frac{{\left(\alpha -r\right)}^{2}}{2\sigma^{2}(\omega +k_{2})}+\frac{2\omega^{2}\beta^{2}\sigma^{2}Q_{2}^{2}(t)}{\omega +k_{2}}+\frac{2(\alpha -r)\omega \beta Q_{2}(t)}{\omega +k_{2}}-2\omega \sigma^{2}\beta^{2}Q_{2}^{2}(t)=0,\;\\ F_{2}(T)=0,\;\\ Q_{2}^{\prime }(t)+Q_{2}^{2}(t)\frac{4\omega k_{2}^{2}\sigma^{2}\beta^{2}}{{\left(\omega +k_{2}\right)}^{2}}+Q_{2}(t)(\frac{\left(2\omega^{2}\beta \sigma^{2}+2k_{2}^{2}\sigma^{2}\beta )(\alpha -r+2k_{2}\sigma^{2}\beta F_{2}(t)\right)}{{\left(\omega +k_{2}\right)}^{2}\sigma^{2}}\;\\ \;-2\alpha \beta -4\sigma^{2}k_{2}\beta^{2}F_{2}(t))+\frac{\omega {\left(\alpha -r+2k_{2}\sigma^{2}\beta F_{2}(t)\right)}^{2}}{{\left(\omega +k_{2}\right)}^{2}\sigma^{2}}=0,\;\\ Q_{2}(T)=0.\; \end{array} & & \end{array}$$ (49)

The optimal investment strategy is


$$\begin{array}{ccc} \begin{array}{cc} \pi^{2*}(t) & =\frac{\left(\alpha -r)+2\sigma^{2}\beta (\omega Q(t)+k_{2}F(t)\right)}{\sigma^{2}s^{2\beta }(\omega +k_{2})e^{-r(T-t)}}. \end{array} & & \end{array}$$ (50)

The investment-only optimization problem has the same investment strategy as the investment-reinsurance problem, which shows that the optimal investment and reinsurance strategies are independent of each other. We can also find that purchasing reinsurance can increase the value function.

**Remark 11.** (GBM Case). If *β* = 0, the CEV model of the problem (16) transforms into the GBM model, the wealth process degenerates to


$$\begin{array}{ccc} \begin{array}{ccc} dX^{3}(t) & =[rX^{3}(t)+(\alpha -r)\pi (t)+(\theta_{1}-\eta_{1})a_{1}+(\theta_{2}-\eta_{2})a_{2}+a_{1}\eta_{1}q_{1}(t)+a_{2}\eta_{2}q_{2}(t) & \\ & \;-\sqrt{\sigma_{1}^{2}q_{1}^{2}(t)+\sigma_{2}^{2}q_{2}^{2}(t)+2q_{1}(t)q_{2}(t)\lambda \mu_{L}\mu_{Y}}\phi_{1}(t)-\sigma \pi (t)\phi_{2}(t)]dt\\ & \;+\sigma \pi (t)dW^{\mathbb{P}*}(t)+\sqrt{\sigma_{1}^{2}q_{1}^{2}(t)+\sigma_{2}^{2}q_{2}^{2}(t)+2q_{1}(t)q_{2}(t)\lambda \mu_{L}\mu_{Y}}dW_{0}^{\mathbb{P}*}(t), \end{array} & & \end{array}$$ (51)

we can quickly get the proposition below.

**Proposition 12.** With respect to the GBM model, the value function is


$$\begin{array}{ccc} \begin{array}{ccc} V^{3}(t,x,s) & =e^{r(T-t)}x+\int_{t}^{T}\{\frac{{\left(\alpha -r\right)}^{2}}{2\sigma^{2}(\omega +k_{2})}-\frac{1}{2(\omega +k_{1})}(\sigma_{1}^{2}+\sigma_{2}^{2} & \\ & \;+2m_{1}m_{2}\lambda \mu_{L}\mu_{Y})+e^{r(T-\nu )}(\theta_{1}-\eta_{1})a_{1}\\ & \;+e^{r(T-\nu )}(\theta_{2}-\eta_{2})a_{2}+\frac{1}{\omega +k_{1}}(a_{1}\eta_{1}+a_{2}\eta_{2})\}d\nu , \end{array} & & \end{array}$$ (52)

the optimal strategy is


$$\begin{array}{lll} \pi^{3*}(t) & =\frac{\alpha -r}{\sigma^{2}(\omega +k_{2})e^{r(T-t)}}, & \\ q_{1}^{3*}(t) & =\frac{m_{1}}{(\omega +k_{1})e^{r(T-t)}},\\ q_{2}^{3*}(t) & =\frac{m_{2}}{(\omega +k_{1})e^{r(T-t)}}, \end{array}$$ (53)

From Proposition 12, we discover that the optimal investment strategy Eq (53) parallels Maenhout’s [19] that studied the optimization problem with the power utility maximization.

## 4 Sensitivity analysis

This section presents some numerical examples to illustrate the effects of some model parameters on the robust equilibrium investment and reinsurance strategy, in which we give some economic explanations. Since we use numerical methods to identify the optimal investment and reinsurance decisions, the input parameter values need to be specified. We use parameter values similar to [2,19,21]. According to the model setting in Sect 2, unless otherwise stated, we select the following parameters throughout this section: risk-free interest rate *r* = 0 . 03, appreciation rate *α* = 0 . 08, volatility coefficients *σ* = 0 . 25, $\sigma_{1}=\sqrt{\left(\lambda_{1}+\lambda )E(L_{i}^{2}\right)}=0.25$, $\sigma_{2}=\sqrt{\left(\lambda_{2}+\lambda )E(Y_{i}^{2}\right)}=0.25$, insurer’s safety loads: $\theta_{1}=0.1$, $\theta_{2}=0.1$, safety loads for claims: $\eta_{1}=0.2$, $\eta_{2}=0.2$, risk-averse coefficient *ω* = 1, ambiguity-aversion coefficients: $k_{1}=0.5$, $k_{2}=0.5$, intensity of Poisson ${\left\{N(t)\right\}}_{t\geq 0}$: *λ* = 1, intensity of Poisson ${\left\{N_{1}(t)\right\}}_{t\geq 0}$: $\lambda_{1}=1$, intensity of Poisson ${\left\{N_{2}(t)\right\}}_{t\geq 0}$: $\lambda_{2}=1$, elasticity parameter *β* = 1 , fixed time horizon *T* = 10, *t* = 0, *x* = 1 . 

**Fig 1:**
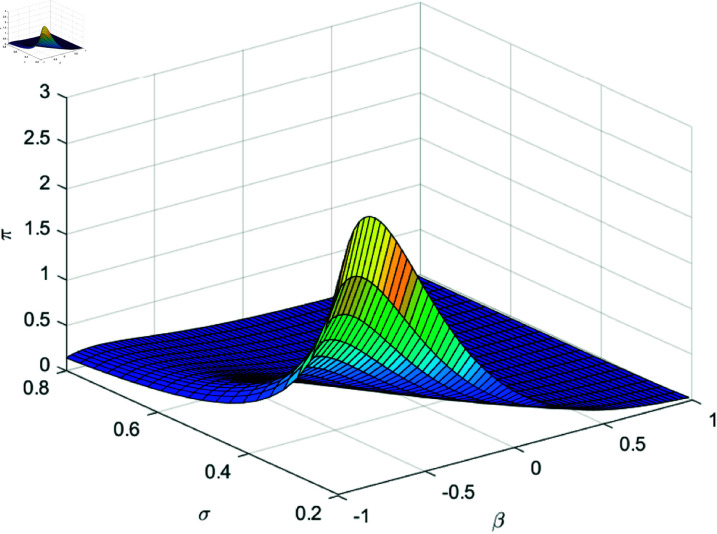
The effect of *σ*, *β* on $\pi^{*}(t)$.

Fig 1 shows the effect of *σ* and *β* on the equilibrium investment strategy. It is easy to find that *π* ( *t* )  decreases with the risky asset volatility *σ* and the elasticity coefficient *β*. In fact, the bigger the value of *σ* is, the larger the instantaneous volatility is, which means the more risk of investment, and thus the insurer should invest less money in the risk asset. In addition, *π* ( *t* )  is a decreasing function in *β*, the larger *β* is, the greater $s^{\beta }$ will be and hence the greater the risk will be; thus, the AAI will wish to invest less in risk asset. Besides, a higher *β* will make the expected volatility drop significantly and increase the probability of adverse changes in risky asset’s price, which means that the increase of the investment risk makes the risk asset more risk and less attractive.

**Fig 2:**
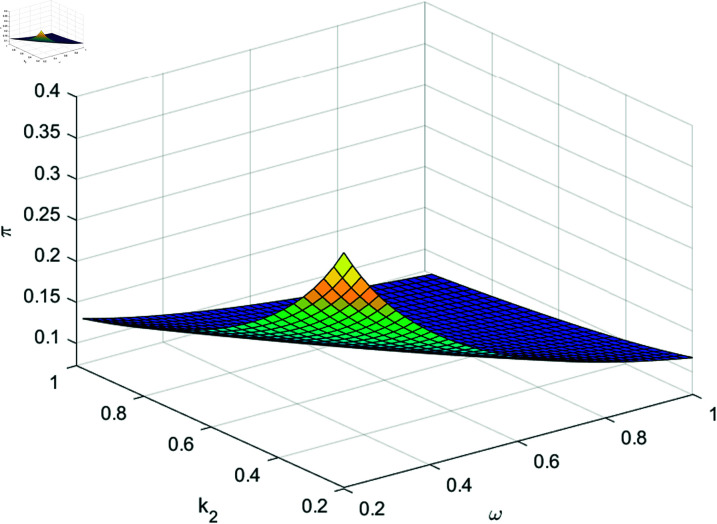
The effect of $k_{2}$, *ω* on $\pi^{*}(t)$.

Fig 2 illustrates that *π* ( *t* )  decreases with $k_{2}$ and *ω*. Recall that $k_{2}$ stands for the AAI’s ambiguity aversion. Larger $k_{2}$ implies that the model uncertainty becomes greater. Hence, the AAI will invest less in the risky asset. Furthermore, from Fig 2, we see that *π* ( *t* )  is a decreasing function with respect to *ω*, since *ω* is the risk-aversion parameter, larger *ω* implies that the less aggressive the AAI will be. Therefore, the AAI will invest less in the risky asset.

**Fig 3:**
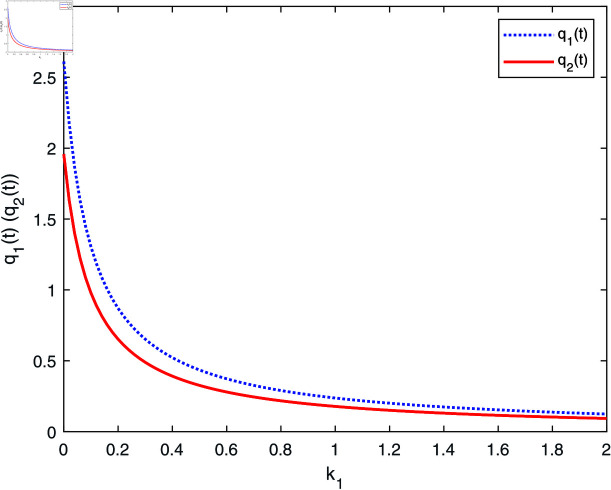
The effect of $k_{1}$ on $q^{*}(t)$.

From Fig 3, we can see that both the retention ratios $q_{1}(t)$ and $q_{2}(t)$ decrease with $k_{1}$, because the higher $k_{1}$ is, the insurer has less confidence in the reference model, and thus the insurer will reduce the retention of $q_{1}(t)$ and $q_{2}(t)$, with the similar mechanism of action to the effects on investment strategy.

**Fig 4:**
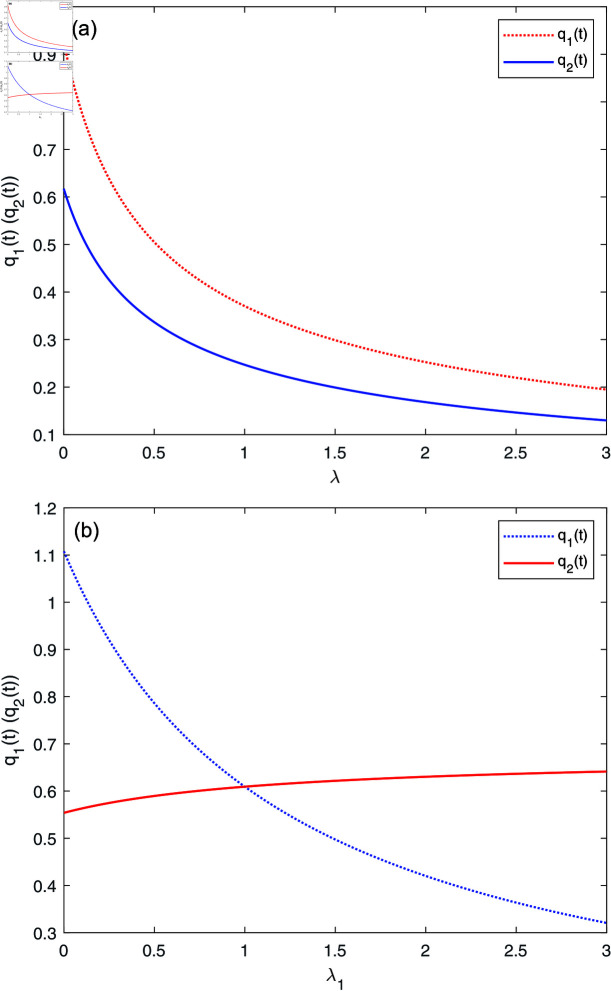
The effects of *λ*, $\lambda_{1}$ on $q^{*}(t)$.

Fig 4 describes the effects of *λ* and $\lambda_{1}$ on reinsurance strategy, respectively. Fig 4(a) shows that the retention ratio $q_{1}(t)$ and $q_{2}(t)$ decrease as *λ* increases. It means that the higher degree of dependence of the two insurance businesses, the greater potential risks of the insurance will face. Thus, the AAI tends to transfer a portion of risk to the reinsurer when the common claim intensity becomes larger. Fig 4(b) reveals that the retention ratio $q_{1}(t)$ decreases with respect to $\lambda_{1}$, whereas $q_{2}(t)$ increases with respect to $\lambda_{1}$. The discoveries make sense because a larger $\lambda_{1}$ represents the claim intensity of the first class business increases, and hence, with a certain overall insurance risk control, the second class business has a comparative advantage due to constant average claims, the AAI prefers to raise the retention level of the second class business and cede more risks of the first class business to the reinsurer.

**Fig 5:**
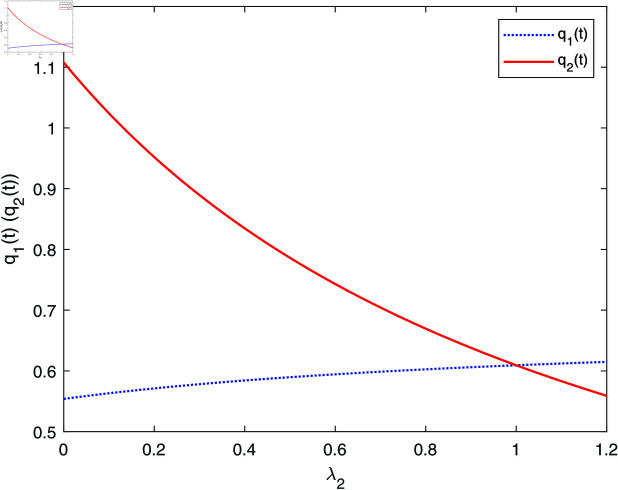
The effect of $\lambda_{2}$ on $q^{*}(t)$.

Fig 5 displays that the retention ratio $q_{2}(t)$ decreases with respect to $\lambda_{2}$, whereas $q_{1}(t)$ increases with respect to $\lambda_{2}$, with an economic explanation similar to that of Fig 4(b).

**Fig 6:**
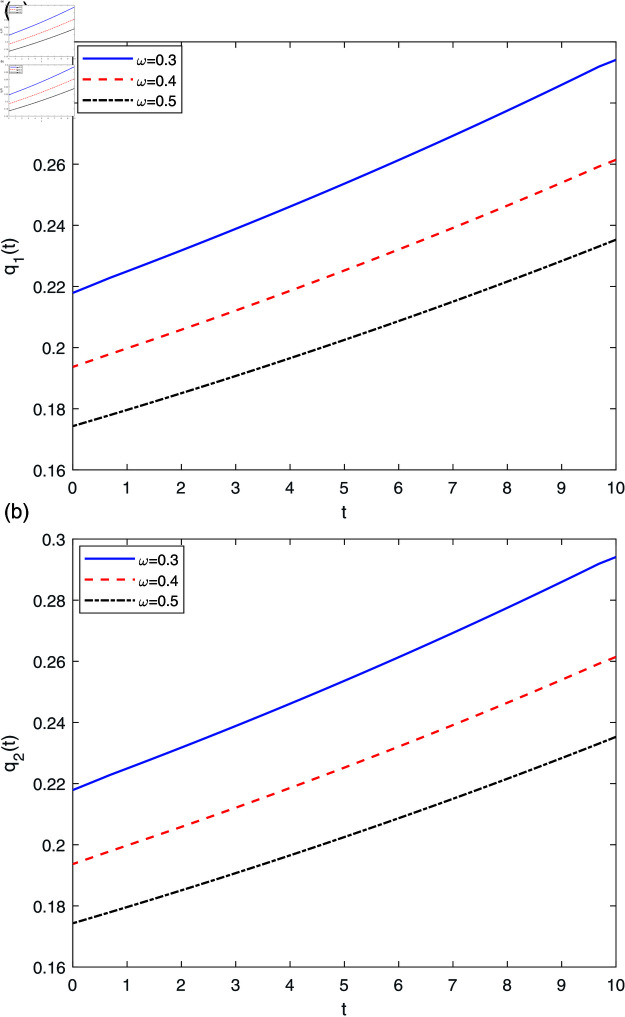
The effect of *ω* on $q^{*}(t)$.

Fig 6 shows that the optimal reinsurance proportion both increase with respect to time *t*, namely, as time elapses, the insurer should keep more insurance business. In addition, Fig 6 depicts the effect of risk-aversion parameter *ω* on the optimal reinsurance strategies. As *ω* increases, both business lines will decrease, that is to say, the more risk-averse the insurer is, the less insurance business the insurer keeps, thus the more reinsurance will be purchased. This can be interpreted as that the presence of ambiguity in the risk profile of insurance surplus is likely to prompt insurers to enhance their dependence on reinsurance as a risk management mechanism.

**Fig 7:**
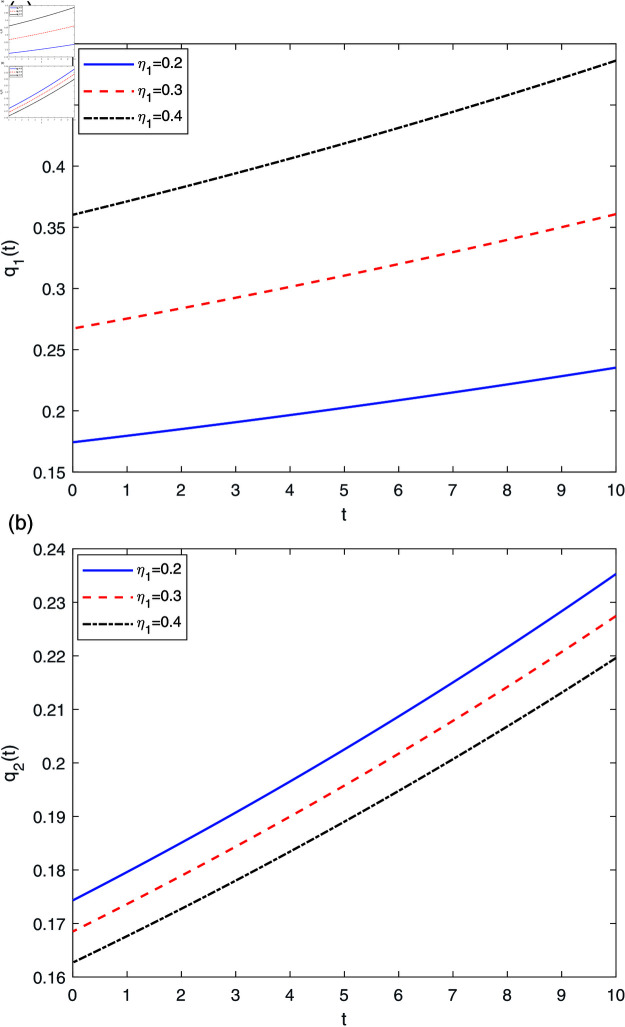
The effect of $\eta_{1}$ on $q^{*}(t)$.

Fig 7 shows the important effects of $\eta_{1}$ on *q* ( *t* ) . From Fig 7, we can see that the AAI prefers to purchase more reinsurance in the short term; however, in the long term, the AAI will buy less reinsurance, especially if the loading factor $\eta_{1}(\eta_{2}$) is high enough, the AAI may not purchase reinsurance. In other words, with the increase of *η*, the AAI tends to accept more insurance businesses in order to reduce the reinsurance cost. These arguments underline the intuitive fact, namely insurers should not rely too much on reinsurance.

**Fig 8:**
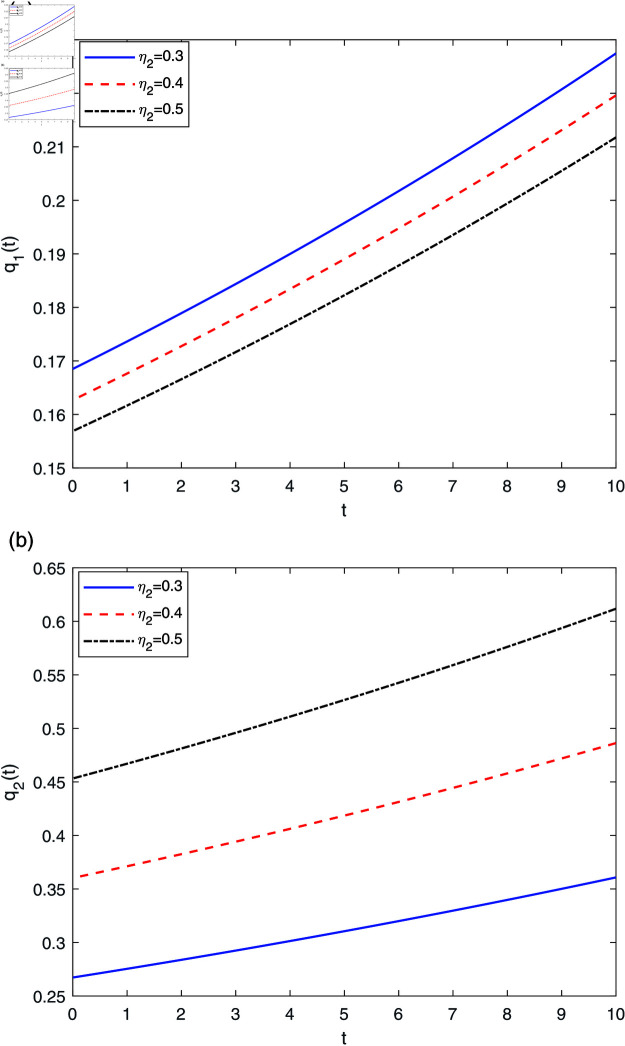
The effect of $\eta_{2}$ on $q^{*}(t)$.

Fig 8 demonstrates the effects of $\eta_{2}$ on reinsurance strategies $q_{1}^{*}(t)$ and $q_{2}^{*}(t)$. In Fig 8, it is easy to see that the retention ratio $q_{1}(t)$ decreases with respect to $\eta_{2}$, whereas $q_{2}(t)$ increases with respect to $\eta_{2}$, with an economic explanation similar to that of Fig 7.

## 5 Conclusion

In this paper, we consider a robust equilibrium investment and reinsurance strategy in a model with stochastic volatility and common shock dependent claims. Firstly, we characterize the insurance market by common shock dependent claims and the financial market by CEV model. Then, we introduce model uncertainty and use it to establish the optimal investment reinsurance problem. Applying the stochastic control theory under the framework of the game theory, we derive a corresponding extended Hamilton–Jacobi–Bellman equation. Furthermore, we use the framework of game theory to solve this problem of time-inconsistency and apply stochastic control theory to seek robust equilibrium strategy. Finally, we give three special cases as well as the economic meaning of numerical examples. The main findings are as follows: (i) The AAI’s attitude towards ambiguity may impact the robust equilibrium investment and reinsurance strategy, such that the optimal investment and reinsurance strategy for the AAI facing model uncertainty is smaller than an ANI. (ii) Compared to investment-only problem, the optimal investment and reinsurance strategy are independent of each other, thus reinsurance increases the optimal value function of the insurer. (iii) The risk common shock factor has a significant impact on all reinsurance business. Therefore, introducing common shocks is more realistic thus making a difference on the equilibrium reinsurance strategy.

There are several potential extensions of this work, such as exploring more sophisticated models, incorporating a mean-variance optimization criterion, and considering non-bankruptcy constraints, dynamic VAR constraints, and dependent risk aversion. However, considering these extensions will make the optimization problem harder. Therefore, the introduction of a novel method to solve the robust optimal investment reinsurance problem has aroused great interest among researchers.

## Supporting information

S1 Appendix. (PDF)
