# Supplementary material for: Time-consistent robust investment-reinsurance strategy with common shock dependence under CEV model
Source: PLoS One. 2025 Feb 28;20(2):e0316649. doi: 10.1371/journal.pone.0316649 (PMC11870388; doi:10.1371/journal.pone.0316649)
Supplement: S1 — (PDF) [file pone.0316649.s001.pdf]

Appendix I. Relative entropy.

The relative entropy of time interval $[t, t+\delta]$ is given by:

$$E^{\mathbb{P}^*}\left[\ln\frac{\Lambda(t+\delta)}{\Lambda(t)}\right] = E^{\mathbb{P}^*}\Big[-\int_t^{t+\delta}\phi_1(u)(dW_0^{\mathbb{P}^*}(u)-\phi_1(u)du)-\tfrac{1}{2}\int_t^{t+\delta}\phi_1^2(u)du$$
$$-\int_t^{t+\delta}\phi_2(u)(dW^{\mathbb{P}^*}(u)-\phi_2(u)du)-\tfrac{1}{2}\int_t^{t+\delta}\phi_2^2(u)du$$
$$=E^{\mathbb{P}^*}\left[\int_t^{t+\delta}\tfrac{1}{2}(\phi_1^2(u)+\phi_2^2(u))du\right].$$

Let $\delta \to 0$, we get the continuous-time limit of the relative entropy. The relative entropy between $\mathbb{P}$ and $\mathbb{P}^*$ is given by

$$[\frac{1}{2}((\phi_1(t))^2+(\phi_2(t))^2)]dt.$$

Appendix II. Proof of Lemma 4.

Using Cauchy-Schwarz inequality, we have

$$(\lambda_1+\lambda)E[L_i^2](\lambda_2+\lambda)E[Y_i^2] \geq \left(\sqrt{(\lambda_1+\lambda)(\lambda_2+\lambda)}E[L_i]E[Y_i]\right)^2 > (\lambda E[L_i]E[Y_i])^2.$$

By $E[L_i^2] > (E[L_i])^2, E[Y_i^2] > (E[Y_i])^2$ and

$$a_1 = (\lambda_1+\lambda)E[L_i], \sigma_1^2 = (\lambda_1+\lambda)E[L_i^2]$$

$$a_2 = (\lambda_2+\lambda)E[Y_i], \sigma_2^2 = (\lambda_2+\lambda)E[Y_i^2]$$

we derive

$$\sigma_1^2\sigma_2^2 > \lambda^2\mu_L^2\mu_Y^2$$

Moreover, we also have

$$\frac{\lambda\mu_L\mu_Y}{\sigma_2^2}\frac{a_2}{a_1} = \frac{\lambda\mu_L\mu_Y}{(\lambda_2+\lambda)\mu_Y'}\frac{(\lambda_2+\lambda)\mu_Y}{(\lambda_1+\lambda)\mu_L} = \frac{\lambda\mu_Y^2}{(\lambda_1+\lambda)\mu_Y'}$$

$$\frac{\sigma_1^2}{\lambda\mu_L\mu_Y}\frac{a_2}{a_1} = \frac{(\lambda_1+\lambda)\mu_L'}{\lambda\mu_L\mu_Y}\frac{(\lambda_2+\lambda)\mu_Y}{(\lambda_1+\lambda)\mu_L} = \frac{(\lambda_2+\lambda)\mu_L'}{\lambda\mu_L^2}$$

Noting that $\mu_L' > \mu_L^2, \mu_Y' > \mu_Y^2$, then we have $\frac{\lambda\mu_Y^2}{(\lambda_1+\lambda)\mu_Y'} < 1$ and $\frac{(\lambda_2+\lambda)\mu_L'}{\lambda\mu_L^2} > 1$. Thus, the lemma holds.

Appendix III. Proof of Theorem 5.

On the basis of the terminal condition of $V(t,x,s)$ and $g(t,x,s)$, we infer the following form:

$$V(t,x,s) = H(t)x + F(t)s^{-2\beta} + G(t), \tag{1}$$
$$g(t,x,s) = P(t)x + Q(t)s^{-2\beta} + R(t), \tag{2}$$

where $H(T) = P(T) = 1$ and $F(T) = Q(T) = 0$. We calculate the derivatives of $V(t,x,s)$ and $g(t,x,s)$ as follows:

$$
\begin{aligned}
&V_t = H'(t)x + F'(t)s^{-2\beta} + G'(t), V_x = H(t), V_s = -2\beta F(t)s^{-2\beta-1},\\
&V_{xx} = 0, V_{ss} = (4\beta^2 + 2\beta)F(t)s^{-2\beta-2}, V_{xs} = 0.\\
&g_t = P'(t)x + Q'(t)s^{-2\beta} + R'(t), g_x = P(t), g_s = -2\beta Q(t)s^{-2\beta-1},\\
&g_{xx} = 0, g_{ss} = (4\beta^2 + 2\beta)Q(t)s^{-2\beta-2}, g_{xs} = 0.
\end{aligned} \tag{3}
$$

Substituting Eqs.(3) into the HJB equation, we have

$$
\begin{aligned}
\sup_{u\in\Pi}\inf_{\phi\in\Phi}\Bigg\{&H'(t)x + F'(t)s^{-2\beta} + G'(t) + [\zeta_1(t) - \zeta_2(t)\phi_1(t)\\
&- \sigma\pi(t)s^{\beta}\phi_2(t)]H(t) - 2\alpha\beta F(t)s^{-2\beta} + 2\sigma\beta F(t)s^{-\beta}\phi_2(t)\\
&- \frac{\omega}{2}(\sigma^2\pi^2(t)s^{2\beta} + \zeta_2^2(t))P^2(t) + (2\beta^2 + \beta)\sigma^2 F(t)\\
&- 2\omega\sigma^2\beta^2 Q^2(t)s^{-2\beta} + 2\omega\beta\sigma^2\pi(t)P(t)Q(t) + \frac{\phi_1^2(t)}{2k_1} + \frac{\phi_2^2(t)}{2k_2}\Bigg\} = 0.
\end{aligned} \tag{4}
$$

Hence we get

$$
\begin{aligned}
&\phi_1^*(t) = k_1\zeta_2(t)H(t),\\
&\phi_2^*(t) = k_2(\sigma\pi(t)s^{\beta}H(t) - 2\sigma\beta F(t)s^{-\beta}).
\end{aligned} \tag{5}
$$

Inserting Eqs.(5) into Eq. (4) yields

$$
\begin{aligned}
\sup_{u\in\Pi}\Bigg\{&H'(t)x + F'(t)s^{-2\beta} + G'(t) + [\zeta_1(t)]H(t) - 2\alpha\beta F(t)s^{-2\beta}\\
&- \frac{\omega}{2}(\sigma^2\pi^2(t)s^{2\beta} + \zeta_2^2(t))P^2(t) + (2\beta^2 + \beta)\sigma^2 F(t) - 2\omega\sigma^2\beta^2 Q^2(t)s^{-2\beta}\\
&+ 2\omega\beta\sigma^2\pi(t)P(t)Q(t) - \frac{k_1(\zeta_2^2(t))H^2(t)}{2}\\
&- \frac{k_2(\sigma\pi(t)s^{\beta}H(t) - 2\sigma\beta F(t)s^{-\beta})^2}{2}\Bigg\} = 0.
\end{aligned} \tag{6}
$$

Differentiating Eq. (6) w.r.t. u implies

$$
\begin{aligned}
&\pi^*(t) = \frac{(\alpha - r)H(t) + 2\sigma^2\beta(\omega P(t)Q(t) + k_2 H(t)F(t))}{\sigma^2 s^{2\beta}(\omega P^2(t) + k_2 H^2(t))},\\
&q_1^*(t) = m_1\frac{H(t)}{\omega P^2(t) + k_1 H^2(t)},\\
&q_2^*(t) = m_2\frac{H(t)}{\omega P^2(t) + k_1 H^2(t)},
\end{aligned} \tag{7}
$$

where $m_1 = \frac{a_1\eta_1\sigma_2^2 - a_2\eta_2\lambda\mu_L\mu_Y}{\sigma_1^2\sigma_2^2 - \lambda^2\mu_L^2\mu_Y^2}$ and $m_2 = \frac{a_2\eta_2\sigma_1^2 - a_1\eta_1\lambda\mu_L\mu_Y}{\sigma_1^2\sigma_2^2 - \lambda^2\mu_L^2\mu_Y^2}$. Substituting Eqs.(7) into

Eqs.(6) and, we get

$$
\begin{aligned}
&[H'(t)+rH(t)]x+s^{-2\beta}\Big\{F^2(t)(-2k_2\sigma^2\beta^2+\frac{2k_2^2\sigma^2\beta^2}{\omega+k_2})\\
&\quad+F(t)(\frac{4\omega\beta^2\sigma^2Q(t)k_2+2(\alpha-r)k_2\beta}{\omega+k_2}-2\alpha\beta)+F'(t)+\frac{(\alpha-r)^2}{2\sigma^2(\omega+k_2)}\\
&\quad+\frac{2\omega^2\beta^2\sigma^2Q^2(t)}{\omega+k_2}+\frac{2(\alpha-r)\omega\beta Q(t)}{\omega+k_2}-2\omega\sigma^2\beta^2Q^2(t)\Big\}\\
&\quad-\frac{H^2(t)}{2(\omega P^2(t)+k_1H^2(t))}(\sigma_1^2+\sigma_2^2+2m_1m_2\lambda\mu_L\mu_Y)\\
&\quad+(2\beta^2+\beta)\sigma^2F(t)+H(t)(\theta_1-\eta_1)a_1+H(t)(\theta_2-\eta_2)a_2\\
&\quad+\frac{H^2(t)}{\omega P^2(t)+k_1H^2(t)}(a_1\eta_1+a_2\eta_2)+G'(t)=0,
\end{aligned}
$$

$$
\begin{aligned}
&[P'(t)+rP(t)]x+s^{-2\beta}\Big\{Q^2(t)\frac{4\omega k_2^2\sigma^2\beta^2}{(\omega+k_2)^2})\\
&\quad+Q(t)(\frac{(2\omega^2\beta\sigma^2+2k_2^2\sigma^2\beta)(\alpha-r+2k_2\sigma^2\beta F(t))}{(\omega+k_2)^2\sigma^2}\\
&\quad-2\alpha\beta-4\sigma^2k_2\beta^2F(t))+Q'(t)+\frac{\omega(\alpha-r+2k_2\sigma^2\beta F(t))^2}{(\omega+k_2)^2\sigma^2}\Big\}\\
&\quad+\frac{a_1\theta_1m_1+a_2\theta_2m_2}{\omega+k_1}+P(t)(\theta_1-\eta_1)a_1+P(t)(\theta_2-\eta_2)a_2\\
&\quad-\frac{k_1}{(\omega+k_1)^2}(\sigma_1^2m_1^2+\sigma_2^2m_2^2+2\lambda\mu_L\mu_Ym_1m_2)+(2\beta^2+\beta)\sigma^2Q(t)+R'(t)=0.
\end{aligned}
$$

Through the separation of variable x and $s^{-2\beta}$, we get the equations as follows:

$$
\begin{cases}
H'(t)+rH(t)=0,\\
F'(t)+F^2(t)(-2k_2\sigma^2\beta^2+\frac{2k_2^2\sigma^2\beta^2}{\omega+k_2})+F(t)(\frac{4\omega\beta^2\sigma^2Q(t)k_2+2(\alpha-r)k_2\beta}{\omega+k_2}-2\alpha\beta)\\
\quad+\frac{(\alpha-r)^2}{2\sigma^2(\omega+k_2)}+\frac{2\omega^2\beta^2\sigma^2Q^2(t)}{\omega+k_2}+\frac{2(\alpha-r)\omega\beta Q(t)}{\omega+k_2}-2\omega\sigma^2\beta^2Q^2(t)=0,\\
-\frac{H^2(t)}{2(\omega P^2(t)+k_1H^2(t))}(\sigma_1^2+\sigma_2^2+2m_1m_2\lambda\mu_L\mu_Y)+(2\beta^2+\beta)\sigma^2F(t)+H(t)(\theta_1-\eta_1)a_1\\
\quad+H(t)(\theta_2-\eta_2)a_2+\frac{H^2(t)}{\omega P^2(t)+k_1H^2(t)}(a_1\eta_1+a_2\eta_2)+G'(t)=0.
\end{cases}
$$

$$
\begin{cases}
P'(t)+rP(t)=0,\\
Q'(t)+Q^2(t)\frac{4\omega k_2^2\sigma^2\beta^2}{(\omega+k_2)^2}+Q(t)(\frac{(2\omega^2\beta\sigma^2+2k_2^2\sigma^2\beta)(\alpha-r+2k_2\sigma^2\beta F(t))}{(\omega+k_2)^2\sigma^2}-2\alpha\beta\\
\quad-4\sigma^2k_2\beta^2F(t))+\frac{\omega(\alpha-r+2k_2\sigma^2\beta F(t))^2}{(\omega+k_2)^2\sigma^2}=0,\\
\frac{a_1\theta_1m_1+a_2\theta_2m_2}{\omega+k_1}+P(t)(\theta_1-\eta_1)a_1+P(t)(\theta_2-\eta_2)a_2-\frac{k_1}{(\omega+k_1)^2}(\sigma_1^2m_1^2+\sigma_2^2m_2^2\\
\quad+2\lambda\mu_L\mu_Ym_1m_2)+(2\beta^2+\beta)\sigma^2Q(t)+R'(t)=0.
\end{cases}
\tag{8}
$$

Considering the boundary conditions $H(T)=1, P(T)=1, F(T)=0, Q(T)=0$, then

$$
H(t)=P(t)=e^{r(T-t)}, \tag{9}
$$

$F(t)$ and $Q(t)$ are decided by

$$\begin{cases} F'(t) + F^2(t)(-2k_2\sigma^2\beta^2 + \frac{2k_2^2\sigma^2\beta^2}{\omega+k_2}) + F(\frac{4\omega\beta^2\sigma^2 Q(t)k_2 + 2(\alpha-r)k_2\beta}{\omega+k_2} - 2\alpha\beta) + \frac{(\alpha-r)^2}{2\sigma^2(\omega+k_2)} \\ \quad + \frac{2\omega^2\beta^2\sigma^2 Q^2(t)}{\omega+k_2} + \frac{2(\alpha-r)\omega\beta Q(t)}{\omega+k_2} - 2\omega\sigma^2\beta^2 Q^2(t) = 0, \\ F(T) = 0, \\ Q'(t) + Q^2(t)\frac{4\omega k_2^2\sigma^2\beta^2}{(\omega+k_2)^2} + Q(t)(\frac{(2\omega^2\beta\sigma^2 + 2k_2^2\sigma^2\beta)(\alpha-r+2k_2\sigma^2\beta F(t))}{(\omega+k_2)^2\sigma^2} - 2\alpha\beta \\ \quad -4\sigma^2 k_2\beta^2 F(t)) + \frac{\omega(\alpha-r+2k_2\sigma^2\beta F(t))^2}{(\omega+k_2)^2\sigma^2} = 0, \\ Q(T) = 0, \end{cases} \tag{10}$$

which has a unique solution, then we obtain the expression of $G(t)$ and $R(t)$:

$$\begin{aligned} G(t) = \int_t^T \Bigg\{ & \frac{-1}{2(\omega+k_1)}(\sigma_1^2 + \sigma_2^2 + 2m_1 m_2 \lambda \mu_L \mu_Y) + (2\beta^2+\beta)\sigma^2 F(\nu) \\ & + e^{r(T-\nu)}(\theta_1 - \eta_1)a_1 + e^{r(T-\nu)}(\theta_2-\eta_2)a_2 + \frac{1}{\omega+k_1}(a_1\eta_1 + a_2\eta_2)\Bigg\} d\nu \end{aligned} \tag{11}$$

$$\begin{aligned} R(t) = \int_t^T \Bigg\{ & P(\nu)(\theta_1-\eta_1)a_1 + P(\nu)(\theta_2-\eta_2)a_2 + \frac{a_1\theta_1 m_1 + a_2\theta_2 m_2}{\omega+k_1} \\ & - \frac{k_1}{(\omega+k_1)^2}(\sigma_1^2 m_1^2 + \sigma_2^2 m_2^2 + 2\lambda\mu_L\mu_Y m_1 m_2) + (2\beta^2+\beta)\sigma^2 Q(\nu)\Bigg\} d\nu. \end{aligned} \tag{12}$$
